# Single-cell RNA sequencing of circulating tumour cells in colorectal cancer

**DOI:** 10.1007/s11033-026-12427-0

**Published:** 2026-07-21

**Authors:** Sai Shyam Vasantharajan, Priyadarshana Ajithkumar, Kit Moloney-Geany, Hannah O’Neill, Euan J. Rodger, Sharon Pattison, Gregory Gimenez, Aniruddha Chatterjee

**Affiliations:** 1 Department of Pathology and Molecular Medicine, Faculty of Medicine, University of Otago, Ōtākou Whakaihu Waka, PO Box 56, Hercus Building, Cnr Great King & Hanover Streets, Otago 9054 Dunedin, New Zealand; 2 Department of Biochemistry, Faculty of Biomedical Sciences, University of Otago, Ōtākau Whakaihu Waka, PO Box 56, 710 Cumberland St, 9016 Dunedin, New Zealand; 3 Health New Zealand Te Whatu Ora – Capital, Coast and Hutt Valley, Private Bag 7902, 49 Riddiford Street, Newtown, 6242 Wellington, New Zealand; 4 School of Health Sciences and Technology, UPES University, P.O. Bidholi, Knowledge Acres, Via Premnagar, Uttarakhand 248007 Dehradun, India; 5 Number 1 Fertility, Melbourne, Australia

**Keywords:** Colorectal Cancer, single-cell RNA sequencing, Epithelial to mesenchymal transition, Hybrid Circulating tumour cell

## Abstract

**Background:**

Circulating tumour cells (CTCs) are key mediators of metastasis and exhibit marked phenotypic plasticity driven by epithelial-to-mesenchymal transition (EMT). Traditional marker-based CTC isolation approaches rely on epithelial marker expression, which may fail to capture mesenchymal and hybrid CTC subpopulations. Hybrid CTCs remain poorly characterised in colorectal cancer (CRC). This study explored transcriptionally defined CTC subpopulations in CRC to provide insight into CTC heterogeneity.

**Methods:**

Single-cell RNA sequencing (scRNA-seq) was performed on peripheral blood mononuclear cell (PBMC) fractions from four treatment-naive CRC patients (AJCC stages I–IV). Integrated analysis with healthy PBMC controls enabled immune cell exclusion and cell-type annotation. CTCs were identified using epithelial and mesenchymal transcriptional scores together with CD45 negativity. Differential expression, pathway enrichment, and pseudotime analyses were used to characterise epithelial, mesenchymal, and hybrid CTC states.

**Results:**

Subpopulations of epithelial, mesenchymal, and hybrid cells were identified in one CRC patient. Hybrid CTCs exhibited distinct transcriptional features and enrichment of pathways related to RNA metabolism, protein trafficking, mitochondrial energy production, DNA repair, and cytoskeletal organisation. Trajectory inference suggested a continuous EMT spectrum, with hybrid CTCs occupying intermediate pseudotime states characterised by progressive loss of epithelial markers and acquisition of mesenchymal-associated features.

**Conclusion:**

This study explored CTC heterogeneity in CRC using single-cell transcriptomics and identified epithelial, hybrid, and mesenchymal CTC states within the analysed sample. Hybrid CTCs exhibited distinct transcriptional features, providing preliminary insight into the transcriptional diversity of CRC CTCs. Further studies in larger cohorts are required to validate these findings and determine their clinical relevance.

**Supplementary Information:**

The online version contains supplementary material available at 10.1007/s11033-026-12427-0.

## Background

Circulating tumour cells (CTCs) are pre-metastatic seeds that enable cancer dissemination [1]. However, the metastatic cascade requires CTCs to execute a series of steps, which include (i) detachment (breaking away from the primary tumour), (ii) migration, (iii) intravasation (entry into the bloodstream), (iv) extravasation (infiltration from the blood vessels to distant tissues), (v) colonisation (adapting to the niche of the organ they metastasise) and, (vi) development of metastatic growth [2, 3]. Therefore, CTCs must be equipped with phenotypic plasticity to adapt to diverse environments encountered during each step of the metastatic process to metastasise successfully [4].

Epithelial-to-mesenchymal transition (EMT) is a driver of phenotypic plasticity in CTCs, resulting in changes such as the loss of Epithelial Cell Adhesion Molecule (EpCAM) expression, enabling CTC formation and survival in the bloodstream [5–7]. EMT-induced plasticity can give rise to heterogeneous CTC subpopulations distributed along a spectrum of epithelial and mesenchymal traits (as EMT is not a binary process [8]). Among these, hybrid CTCs that partially retain epithelial and mesenchymal features may have a selective advantage, as they can switch between EMT and MET states, enhancing both survival in circulation and colonisation potential [9]. Studies have shown that the presence of mesenchymal and hybrid CTC phenotypes is closely associated with poorer prognosis in breast, liver and colorectal cancers [10–14].

However, current marker-based methods for CTC isolation, such as CellSearch, rely on epithelial markers like EpCAM for CTC isolation. As a result, these methods cannot detect CTCs that do not express these markers, thereby failing to capture heterogeneity within the CTC population [15, 16]. This reduces the repertoire of CTCs available for downstream analysis, limiting insights into the metastatic process. Capturing diverse CTC subpopulations is therefore crucial to better understand CTC-mediated colorectal cancer (CRC) metastasis.

The advent of single-cell RNA sequencing (scRNA-seq) technologies has provided a high-throughput platform for investigating heterogeneity in CTC populations, as it can generate transcriptomes at the single-cell level without relying solely on predefined epithelial markers for CTC identification. A study in 2021 profiled CTCs from CRC patients using the Switching Mechanism at the 5’ End of RNA Transcript (SMART-seq) technology and demonstrated substantial heterogeneity within CTC populations, with epithelial, mesenchymal, and stem cell-related transcriptional programmes identified across individual CTCs [10, 17]. These findings underscore the potential of single-cell approaches for detecting non-epithelial CTC subpopulations in CRC patients that can be missed by marker-based methods. However, this study examined CTC heterogeneity solely in late‑stage CRC.

Additionally, to the best of our knowledge, scRNA-seq studies of CRC CTCs, such as the study by Kozuka et al. [10], have primarily focused on detecting and classifying CTCs based on epithelial, mesenchymal, or stem cell-related programs. However, this means that hybrid CTCs, which exhibit both epithelial and mesenchymal traits, have been less explored. In this study, we aimed to address this gap by applying high-throughput 10X Genomics scRNA-seq to peripheral blood mononuclear cells (PBMCs) from CRC patients in different CRC stages. The goal was to identify and characterise hybrid CTC populations, which are emerging as subpopulations with higher metastatic potential, and investigate the transcriptional programmes that may support their survival and dissemination.

## Methods

**Fig. 1:**
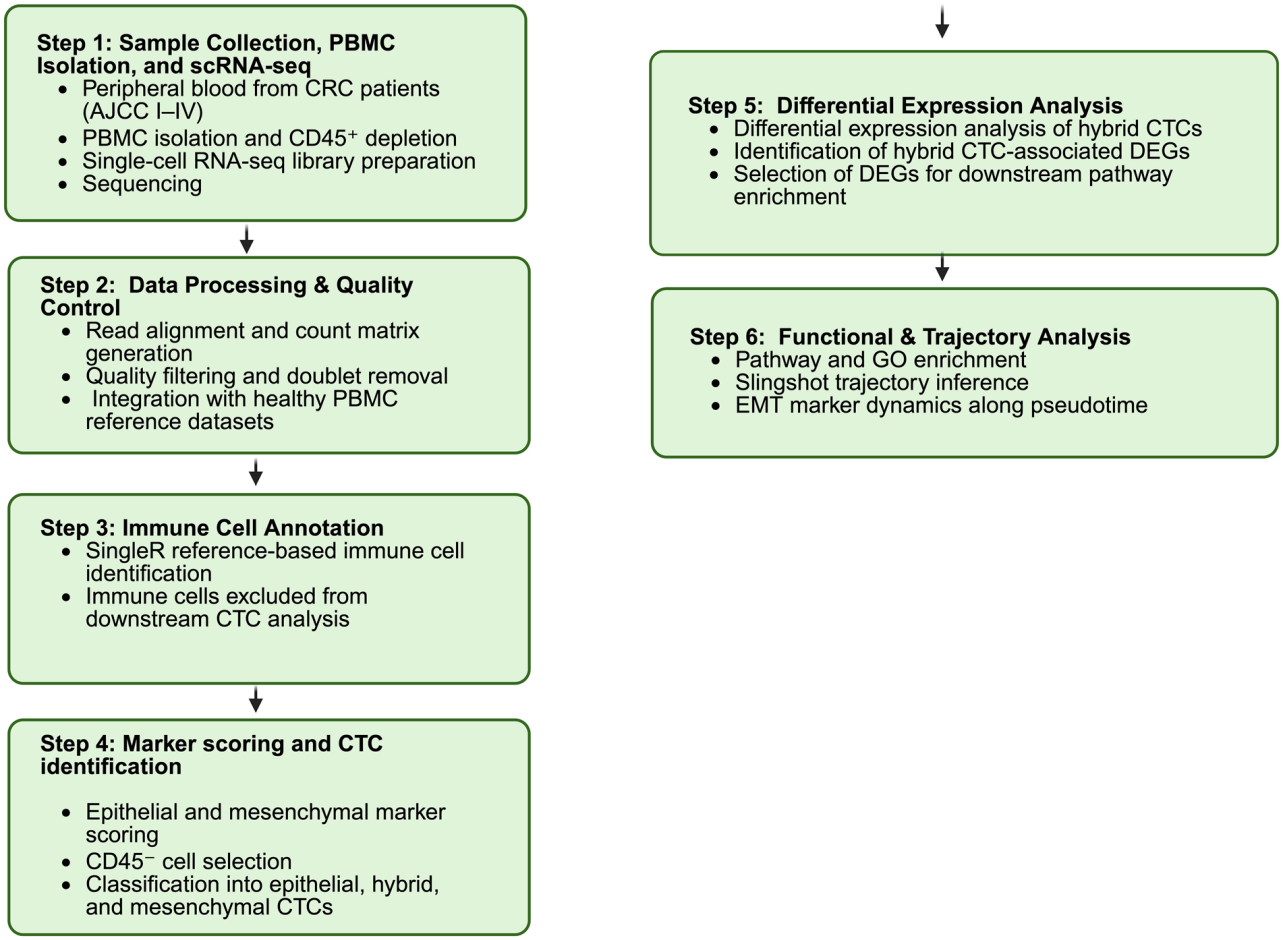
Overview of the experimental and analytical workflow for single-cell RNA-seq–based identification and analysis of CTCs. Created in Biorender.com

An overview of the experimental and analytical workflow is shown in Fig. 1.

### Ethics, Patient Selection, and Clinical Characteristics

This study was approved by the Health and Disability Ethics Committee, New Zealand (HDEC; reference #14/NTA/33). Written informed consent was obtained from all participants. Peripheral blood samples were collected prior to surgery from four treatment-naive CRC patients, representing stages I–IV (classification based on AJCC 8th edition [18]). Patient characteristics are summarised in Table 1.

**Table 1:** Characteristics of patients used for scRNA-seq

Sample ID	Gender	Age (Yrs)	AJCC stage
CRC_1	M	87	II
CRC_2	M	44	IV
CRC_3	F	85	III
CRC_4	M	72	I

**Abbreviations**: **Yrs -** years, **AJCC -** American Joint Committee on Cancer

### Single-Cell cDNA Library Preparation and Sequencing

PBMCs were isolated from 10 mL of blood collected from four CRC patients (AJCC stages I–IV; Table 1) prior to surgery. PBMC isolation was performed using Lymphoprep (MediRay, Cat# N1114544), followed by depletion of CD45 + cells with the EasySep™ Human CD45 Depletion Kit II (Cat# 17898, StemCell Technologies) to enrich for non-haematopoietic populations. Single-cell cDNA libraries were prepared from 3000–9000 PBMCs per sample using the Chromium Next GEM Single Cell 3’ Kit v3.1 (10X Genomics, Cat#10X-1000269) according to the manufacturer’s protocol [19].

Following cell capture, lysis, cDNA synthesis, and amplification (14 cycles), 25 ng of cDNA from each sample was used for library construction. Libraries were assessed for quality on a Bioanalyser (Agilent 2100), pooled, and sequenced on a NextSeq2000 at the Otago Genomics Facility, New Zealand following 10X Genomics recommendations (paired-end, dual indexing, Read 1: 28 cycles, i7 index: 8 cycles, i5 index: 0 cycles, Read 2: 90 cycles).

As controls, publicly available scRNA-seq datasets of healthy PBMCs from volunteers (GEO: GSM5335490 and GSM5335491) were included [20]). These datasets were generated using the Chromium Single Cell 3ʹ GEM, Library & Gel Bead Kit v3 (16 rxns, PN-1000075; 10x Genomics) and were processed identically as the CRC samples for integration and batch correction [21].

### Single-Cell RNA-Seq Data Preprocessing and Integration

Raw sequencing reads for the CRC samples were processed with Cell Ranger v6.0.1 to generate gene–cell count matrices [19]. Gene-cell count matrices for the healthy controls were downloaded from the GEO database. Seurat v5.3.1 was used for quality control, dataset integration, clustering, and cell-type annotation [22]. Prior to quality-control filtering and doublet removal, additional analyses examining EPCAM-positive, epithelial, mesenchymal, and CD45-associated cell populations across sequential preprocessing stages were performed (see Supplementary Methods section Assessment of epithelial, mesenchymal, and CD45 cell populations across preprocessing stages). Cells with fewer than 200 detected genes, more than 2,500 detected genes, or greater than 20% mitochondrial reads were removed [23]. Genes expressed in fewer than three cells were excluded, and doublets were identified and removed using scDblFinder [24].

CRC and healthy control datasets were integrated using Seurat’s anchor-based integration workflow (see Supplementary Methods section Integration of CRC and healthy control datasets). Batch-correction performance was subsequently evaluated using UMAP visualisation together with centroid distance and Local Inverse Simpson’s Index (LISI) analyses to assess sample mixing while preserving broader biological structure (see Supplementary Methods section Assessment of batch correction and dataset integration) [25]. Healthy PBMC datasets were included solely to support immune-cell annotation and define reference expression patterns for non-tumour cells. As disease status and dataset origin were fully confounded, direct quantitative comparisons between CRC and control cells were not performed. Accordingly, all downstream analyses related to CTC identification, subtyping, differential expression, pathway enrichment, and trajectory inference were restricted to CRC-derived cells. The batch-corrected RNA expression matrix was extracted from the integrated dataset for downstream analyses.

### Marker Scoring, Immune Annotation, and CTC Identification

Per-cell epithelial, mesenchymal, and CD45 scores were calculated from the integrated, log-normalised RNA expression matrix. For epithelial and mesenchymal states, scores were computed by summing the normalised expression values of predefined marker genes curated from prior literature (Table S1).

Only marker genes detected in the dataset were included, and no additional weighting or scaling was applied. The expression patterns of the selected epithelial and mesenchymal marker genes are shown in Fig S1. Epithelial and mesenchymal scores were calculated using unweighted summation of marker expression values, consistent with prior single-cell EMT studies that apply unweighted marker aggregation to capture continuum-like transcriptional states [26–28]. Additional details regarding the assessment of marker-level expression patterns are provided in the Supplementary Methods section Assessment of Marker-Level Expression Patterns.

Because PBMC preparations contain multiple haematopoietic lineages, *CD45* expression alone was insufficient for reliable immune cell identification. Immune cells were therefore identified using SingleR with the Human Primary Cell Atlas reference. Each cell was assigned to its closest reference population, and cells annotated as canonical immune lineages (T cells, B cells, natural killer cells, monocytes, dendritic cells, granulocytes, macrophages, platelets, or erythroid cells) were labelled as Immune. This immune annotation was applied prior to CTC classification and took precedence over all marker-based classifications, ensuring that immune cells were not misclassified as tumour-like.

CTC identification was restricted to CRC patient-derived cells. For these cells, epithelial and mesenchymal score thresholds were defined empirically as the 75th percentile of the respective score distributions within CRC samples. This quantile-based approach was used to identify transcriptionally extreme cells relative to the patient background, rather than applying an absolute expression cut-off. CD45-negative status was required for CTC identification and was defined as *CD45* expression ≤ 0.1. This threshold was determined by inspecting the *CD45* expression distributions across all samples, including controls, where values below 0.1 corresponded to a distinct low-expression population while allowing for low-level technical noise. *CD45* expression distributions and the selected threshold are shown in Fig S2. Following threshold definition, a cell was classified as CTC only if it met all of the following criteria: (i) originated from a CRC sample, (ii) exceeded the epithelial or mesenchymal score threshold, (iii) had *CD45* expression ≤ 0.1, and (iv) was not annotated as immune by SingleR. CTCs were further subclassified as epithelial, mesenchymal, or hybrid depending on whether epithelial scores alone, mesenchymal scores alone, or both scores exceeded their respective thresholds.

To assess the stability of CTC classification across different scoring thresholds, epithelial and mesenchymal upper-quantile cut-offs were systematically varied from 50% to 95%, and the resulting number of classified cells was recorded at each threshold. This approach is consistent with EMT-based scoring strategies applied in CTC studies and informed selection of thresholds for downstream CTC classification [29].

To generate a single biologically consistent label per cell, immune labels took precedence over all other classifications. Among non-immune cells, CTC subtype labels were retained, and remaining cells were categorised as non-CTC PBMCs. This hierarchical strategy provided a stable framework for downstream analysis and visualisation (Fig S3). UMAP coordinates were extracted from the integrated object and linked with combined labels to maintain consistent cell annotation across the dataset. *CD45* expression was examined across combined labels to assess immune and non-immune classification.

### Cell Annotation and UMAP Visualisation

Following marker scoring, immune annotation, and CTC classification, all cells were assigned a single, unified label (“CombinedLabel”) to facilitate downstream analysis. Immune subtypes were first annotated using SingleR outputs by mapping to canonical immune lineages (T cells, B cells, Natural killer cells, monocytes, dendritic cells, granulocytes, macrophages, platelets, and erythroid cells). Next, CTC type labels (epithelial, mesenchymal, hybrid) were overlaid for non-immune cells, and any remaining cells were labelled as non-CTC/non-immune PBMCs, maintaining a biologically consistent hierarchical annotation.

To visualise cellular heterogeneity, UMAP embeddings were extracted from the integrated object, merged with CombinedLabel metadata, and plotted with standardised axis naming to ensure reproducibility [30]. Distinct colour codes were assigned to each cell type, including CTC subtypes and immune populations, allowing visual assessment of transcriptional patterns across cells.

### CTC Subsetting, Differential Expression, Visualisation, and Pathway Enrichment

CTCs were first extracted from the integrated Seurat object by selecting cells annotated as CTCs in the metadata. Only cells with a valid *CTC_type* annotation were retained for downstream analyses. Based on established EMT transition marker signatures [29], CTCs were classified into three transcriptional states: epithelial, hybrid, and mesenchymal. For each CTC subtype, the number of cells and the corresponding patient samples were assessed. Differential gene expression between CTC subtypes was performed at the single-cell level using Seurat’s Wilcoxon rank sum test with default settings, a log2 fold-change threshold of 0.25, and a minimum detection threshold of 10% [22].

To visualise subtype-specific transcriptional patterns, the top 100 differentially expressed genes per subtype, ranked by log2 fold change, were selected from the per-cell results. Normalised expression values were extracted from the Seurat object, scaled by gene, and ordered by CTC subtype. Heatmaps were generated using *pheatmap* with genes clustering and fixed cells ordering corresponding to epithelial, hybrid, and mesenchymal CTCs. CTC subtype and patient sample information were included as column annotations.

To specifically characterise transcriptional programmes associated with hybrid CTCs, hybrid cells were compared against non-hybrid CTCs (epithelial and mesenchymal CTCs combined). Differential expression was performed using the default settings of Seurat’s *FindMarkers* function with logfc.threshold set to 0, allowing all genes to be evaluated initially, including those with small fold changes. Genes were considered as significantly differentially expressed based on an adjusted p-value < 0.05 and an absolute log₂ fold change > 0.25.

While significant genes were identified and reported, pathway enrichment analyses were performed using the full set of genes tested in the differential expression analysis to capture coordinated pathway-level transcriptional changes. Genes were converted to Entrez IDs using the *clusterProfiler bitr* function [31]. Enrichment of Gene Ontology Biological Process (GOBP) terms was performed using *enrichGO* with Benjamini-Hochberg correction. *KEGG* pathway enrichment was performed using enrichKEGG with default human annotations.

### CTC Trajectory and Pseudotime Analysis

To investigate transcriptional continuity across CTC states, trajectory inference was performed using Slingshot [32]. Analyses were restricted to cells classified as CTCs. A *SingleCellExperiment* object was constructed using the integrated, log-normalised RNA expression matrix. Slingshot was chosen because it performs lineage inference at the cluster level, which reduces the impact of technical noise and dropout compared with cell-to-cell distance–based methods. This is particularly relevant for rare cell populations such as CTCs, where individual cells may be exhibit transcriptional ambiguity. In addition, Slingshot allows trajectories to be inferred without requiring strong a priori assumptions about the number, order, or identity of terminal states, while still supporting biologically interpretable lineage structures.

UMAP dimensionality reduction was recomputed specifically for trajectory analysis using the *uwot* package (15 nearest neighbours, minimum distance = 0.1), a commonly used parameterisation that balances preservation of local neighbourhood structure with global continuity in scRNA-seq data. CTC subtype labels (epithelial, hybrid, mesenchymal) were used as cluster annotations for trajectory inference. Slingshot was applied using UMAP coordinates without specifying an initial or terminal state. Pseudotime values were then extracted from the first inferred lineage and used for downstream visualisation. Cells were visualised on UMAP embeddings coloured by pseudotime and CTC subtype, with inferred trajectory curves overlaid.

To examine marker dynamics along pseudotime, predefined epithelial and mesenchymal marker genes were selected. Expression values were extracted from the log-normalised matrix, cells were ordered by pseudotime, and gene expression values were scaled per gene. Heatmaps were generated with fixed gene ordering and without clustering to highlight gradual changes in marker expression along the inferred trajectory.

## Results

### Quality control and batch correction of single-cell RNA-seq data

Cell Ranger analysis identified between 3,025 and 9,130 cells across the CRC and control datasets prior to filtering (Fig S4). Filtering removed cells with < 200 detected genes, > 2500 genes, high mitochondrial content (> 20%), and potential doublets. Following filtering, between 1,635 and 5,073 cells remained for downstream analysis (Fig S4).

Assessment of raw count matrices identified between 1,363 and 5,087 CD45-positive cells across the CRC and control datasets (Fig S5a). Four EpCAM-positive cells were detected in Control_2, whereas no EpCAM-positive cells were detected in the remaining samples (Fig S5a). Following mitochondrial filtering, reductions in epithelial, mesenchymal, and CD45-associated cell populations were observed across all samples (Fig S5b). Further reductions in these populations were observed following doublet removal, although epithelial, mesenchymal, and CD45-associated populations remained detectable across all preprocessing stages (Fig S5c).

**Fig. 2:**
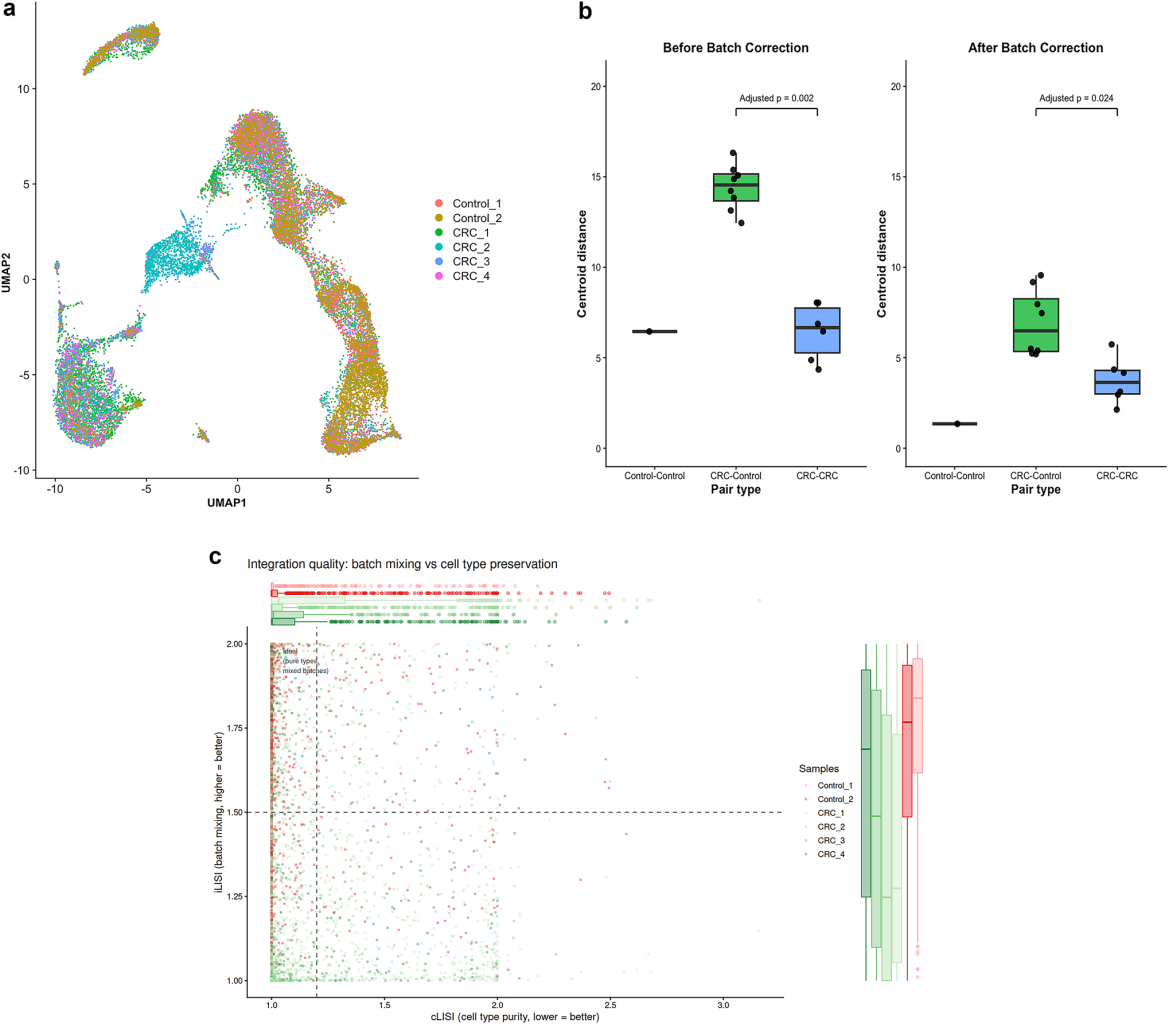
Assessment of batch effects and sample mixing in scRNA-seq data. **(a)** UMAP projection of scRNA-seq data following batch correction. Each dot represents an individual cell, and colours indicate the sample source of each cell (Control_1, salmon pink; Control_2, mustard yellow; CRC_1, green; CRC_2, cyan; CRC_3, blue; CRC_4, magenta). UMAP1 and UMAP2 represent the first and second UMAP dimensions. **(b)** Comparison of pairwise centroid distances between Control–Control, CRC–CRC, and CRC–Control sample groups before (left) and after (right) batch correction. The x-axis represents sample pair type and the y-axis represents centroid distance calculated in PCA space. Adjusted p-values are shown above the plots. **(c)** Local inverse Simpson’s index (LISI) analysis following batch correction. The central scatterplot shows cLISI scores on the x-axis and iLISI scores on the y-axis. Distribution plots above and to the right summarise the distributions of cLISI and iLISI scores, respectively

Following preprocessing, potential batch effects between the CRC datasets and healthy control datasets were assessed. Prior to batch correction, cells clustered predominantly according to sample of origin (Fig S6a). Following batch correction, improved sample mixing was observed in the integrated UMAP (Fig. 2a; Fig S6b). Pairwise centroid distance analysis demonstrated reduced inter-sample separation following batch correction (Fig. 2b), while Local inverse Simpson’s index (LISI) analysis indicated improved batch mixing while retaining broader biological structure (Fig. 2c). The integrated dataset were clustered at a resolution of 0.8, generating 20 clusters for downstream visualisation and analysis.

### Candidate CTC enrichment and immune-cell exclusion

Building on the integrated dataset, we applied a targeted marker-guided enrichment workflow to identify CTC-like cells while excluding immune contaminants. Immune cells were identified using a combined approach, including SingleR annotation and *CD45* expression (> 0.1), ensuring capture of canonical immune populations.

Candidate CTCs were identified by screening epithelial, mesenchymal and hybrid transcriptional programmes using a curated multi-marker panel and by excluding CD45 + cells. Programme scores were calculated as the column-sum of log-normalised expression across the marker lists, and cells exceeding the 0.75 quantile for either epithelial or mesenchymal scores (calculated across CRC samples) were classified as candidate CTCs. Assessment across different quantile cut-offs showed that epithelial candidate counts remained stable until very high cutoffs (0.90), whereas mesenchymal candidate counts declined markedly with stricter cut-offs (0.95), supporting selection of the 0.75 quantile (Table S2).

**Fig. 3:**
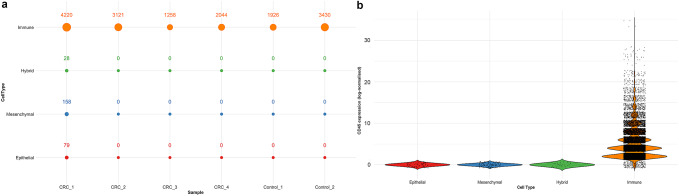
Identification of CTC-like cells and confirmation of immune-cell exclusion. **(a)** Dot plot showing the distribution of epithelial (red dots), mesenchymal (blue dots), hybrid (green dots), and immune populations (orange dots) across all CRC and control samples. The numbers above the dots correspond total numbers of cells belonging to respective cell type in each sample. Candidate CTC subsets (epithelial, mesenchymal, and hybrid) are enriched only in CRC_1, while other samples contain predominantly immune PBMC populations. **(b)** Violin plot of *CD45* expression across major cell groups (epithelial, mesenchymal, hybrid, and immune). Immune PBMCs show high *CD45* expression, whereas epithelial, mesenchymal, and hybrid candidate CTCs display minimal *CD45* expression, confirming effective exclusion of immune contaminants during CTC enrichment

Only CRC_1 contained identifiable candidate CTCs (*n* = 265), which comprised Epithelial (*n* = 79), Hybrid (*n* = 28) and Mesenchymal (*n* = 158) subsets (Fig. 3a). Following immune exclusion (Fig. 3b), the CTC-enriched population was used for downstream dimensionality reduction, clustering and differential expression analyses. The different CTC states were distributed throughout the UMAP rather than forming distinct clusters (Fig S7), reflecting the transcriptional heterogeneity of the CTC population.

Of the 24 epithelial markers included in the panel, four were detected in CRC_1 CTCs (Table S3), with *KRT10* showing the highest expression, followed by *CDH1*, *KRT5*, and *KRT1*. Of the 24 mesenchymal markers, 14 were detected, with *MALAT1* showing the highest expression, followed by *S100A4* and *VIM*. Markers not detected in the expression matrix are reported as “Not detected” (Table S3).

### Gene expression patterns in hybrid CTCs

**Fig. 4:**
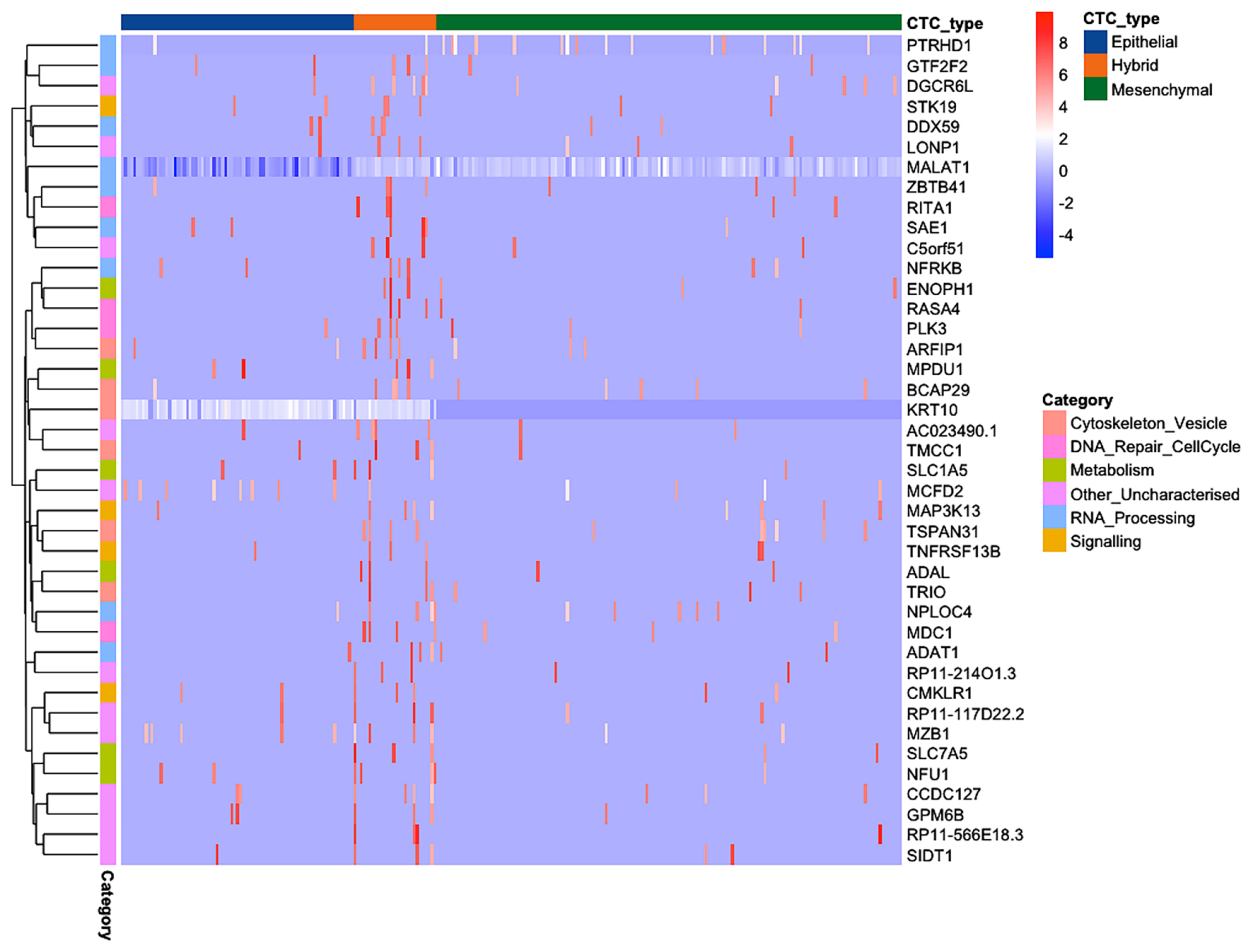
Expression of 41 key functional genes across CTC subtypes. Heatmap showing scaled RNA expression of 41 genes selected based on differential expression in hybrid CTCs compared to epithelial and mesenchymal CTCs. Rows represent individual genes. Expression values are log normalised values per gene (row). Columns represent individual cells, ordered by CTC subtype (*Epithelial*, *Hybrid*, *Mesenchymal*), with distinct column colours indicating subtype. Hierarchical clustering was applied to genes, while cell order was fixed by subtype. Genes related to *Metabolism*, *DNA repair*,* cell cycle*, *Cytoskeleton vesicle trafficking*, *RNA processing*, and *Signalling* were found to be upregulated in hybrid CTCs

The top 100 differentially expressed genes were examined using scaled expression values from the integrated dataset (Fig. S8), of which 41 were upregulated in hybrid CTCs relative to epithelial and mesenchymal CTCs (Fig. 4).

Metabolic genes upregulated in hybrid CTCs included Solute Carrier Family 1 Member 5 (*SLC1A5*), Solute Carrier Family 7 Member 5 (*SLC7A5*), and Enolase-Phosphatase 1 (*ENOPH1*). Genes associated with DNA repair and cell cycle regulation included Mediator of DNA Damage Checkpoint 1 (MDC1), Polo Like Kinase 3 (*PLK3*), RAS P21 Protein Activator 4 (*RASA4*), and RBPJ Interacting and Tubulin Associated 1 (*RITA1*).

Hybrid CTCs also showed increased expression of genes involved in actin cytoskeleton organisation and vesicle trafficking, including ADP Ribosylation Factor Interacting Protein 1 (*ARFIP1*), Triple Functional Domain Protein (*TRIO*), B-Cell Receptor Associated Protein 29 (*BCAP29*), and Tetraspanin 31 (*TSPAN31*). Genes involved in RNA processing and translation included DEAD-Box Helicase 59 (*DDX59*), Adenosine Deaminase Acting on tRNA 1 (*ADAT1*), and General Transcription Factor IIF Subunit 2 (*GTF2F2*).

Regulatory and signalling genes showing increased expression in hybrid CTCs included Serine/Threonine Kinase 19 (*STK19*), TNF Receptor Superfamily Member 13B (*TNFRSF13B*), Chemokine Like Receptor 1 (*CMKLR1*), Zinc Finger And BTB Domain Containing 41 (*ZBTB41*), Nuclear Factor Related To Kappa B Binding Protein (*NFRKB*), SUMO1 Activating Enzyme Subunit 1 (*SAE1*), and NPL4 Homolog, Ubiquitin Recognition Factor (*NPLOC4*).

Among canonical lineage markers, *KRT10* was upregulated in epithelial and hybrid CTCs but absent in mesenchymal CTCs, whereas *MALAT1* showed elevated expression in hybrid and mesenchymal CTCs but was not detected in epithelial CTCs (Fig. 4).

### Pseudotime analysis of epithelial, hybrid, and mesenchymal CTCs

**Fig. 5:**
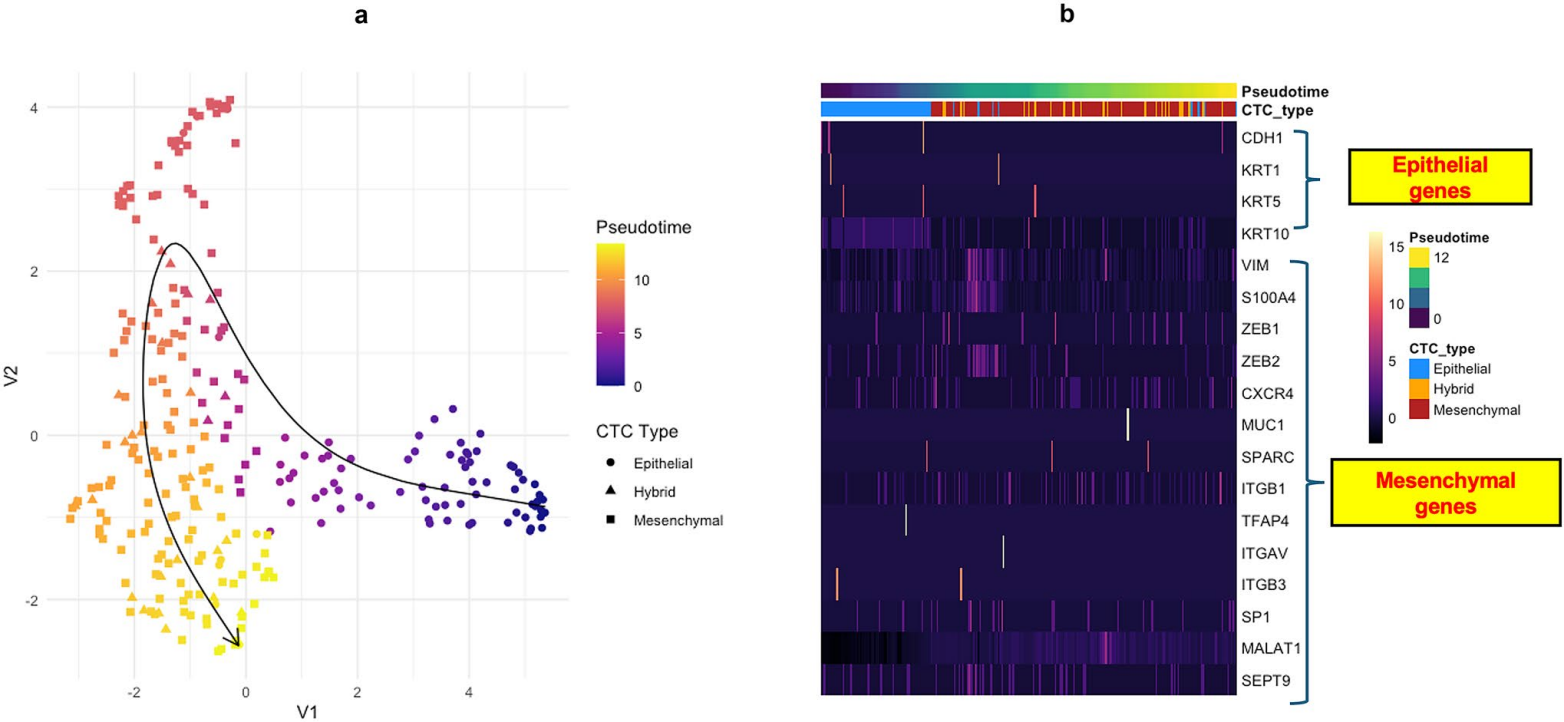
Pseudotime analysis of epithelial, hybrid, and mesenchymal CTC states. **(a)** UMAP visualisation of epithelial, hybrid, and mesenchymal CTCs coloured by inferred pseudotime following trajectory inference using Slingshot. Each point represents a single CTC, with epithelial CTCs shown as circles, hybrid CTCs as triangles, and mesenchymal CTCs as squares. Pseudotime is indicated by a colour gradient, with dark blue representing low pseudotime and yellow representing high pseudotime. Cells at low pseudotime are predominantly epithelial and appear relatively homogeneous, whereas increasing pseudotime is associated with the emergence of hybrid CTCs followed by mesenchymal CTCs and increased transcriptional heterogeneity. The black arrow indicates the inferred direction of the EMT continuum. UMAP dimensions V1 and V2 represent the first two components of the low-dimensional embedding used for visualisation. **(b)** Heatmap showing scaled expression of selected epithelial and mesenchymal marker genes ordered by inferred pseudotime. Rows represent individual genes and columns represent single CTCs arranged from low to high pseudotime. CTC subtype annotation is shown above the heatmap, with epithelial, hybrid, and mesenchymal CTCs indicated by blue, yellow, and maroon colours, respectively. The pseudotime bar shows the same colour gradient as in panel (a), with dark blue indicating low pseudotime and yellow indicating high pseudotime. *KRT10* expression decreases progressively along pseudotime, while *MALAT1* expression increases, consistent with a gradual transition from epithelial to mesenchymal transcriptional states

To investigate transcriptional continuity across CTC states, trajectory inference was performed using Slingshot. When visualised on the UMAP, CTCs were ordered along a single dominant trajectory, with epithelial CTCs enriched at low pseudotime (Fig. 5a), hybrid CTCs occupying intermediate positions, and mesenchymal CTCs predominantly located at high pseudotime. Increasing transcriptional heterogeneity was observed along the trajectory, consistent with the progressive phenotypic diversification that occurs during EMT.

To examine marker dynamics along pseudotime, epithelial and mesenchymal marker genes were visualised in a scaled heatmap ordered by inferred pseudotime. Among epithelial markers, *KRT10* showed high expression in epithelial CTCs, was retained in hybrid cells, and gradually decreased toward mesenchymal CTCs (Fig. 5b). In contrast, the mesenchymal-associated transcript *MALAT1* displayed low expression at early pseudotime and increased progressively toward late pseudotime (Fig. 5b). Other epithelial and mesenchymal markers exhibited more variable patterns but broadly aligned with this transition. Together, these results indicate a continuous transcriptional spectrum linking epithelial, hybrid, and mesenchymal CTC states.

### Pathway enrichment analysis of hybrid CTCs

**Fig. 6:**
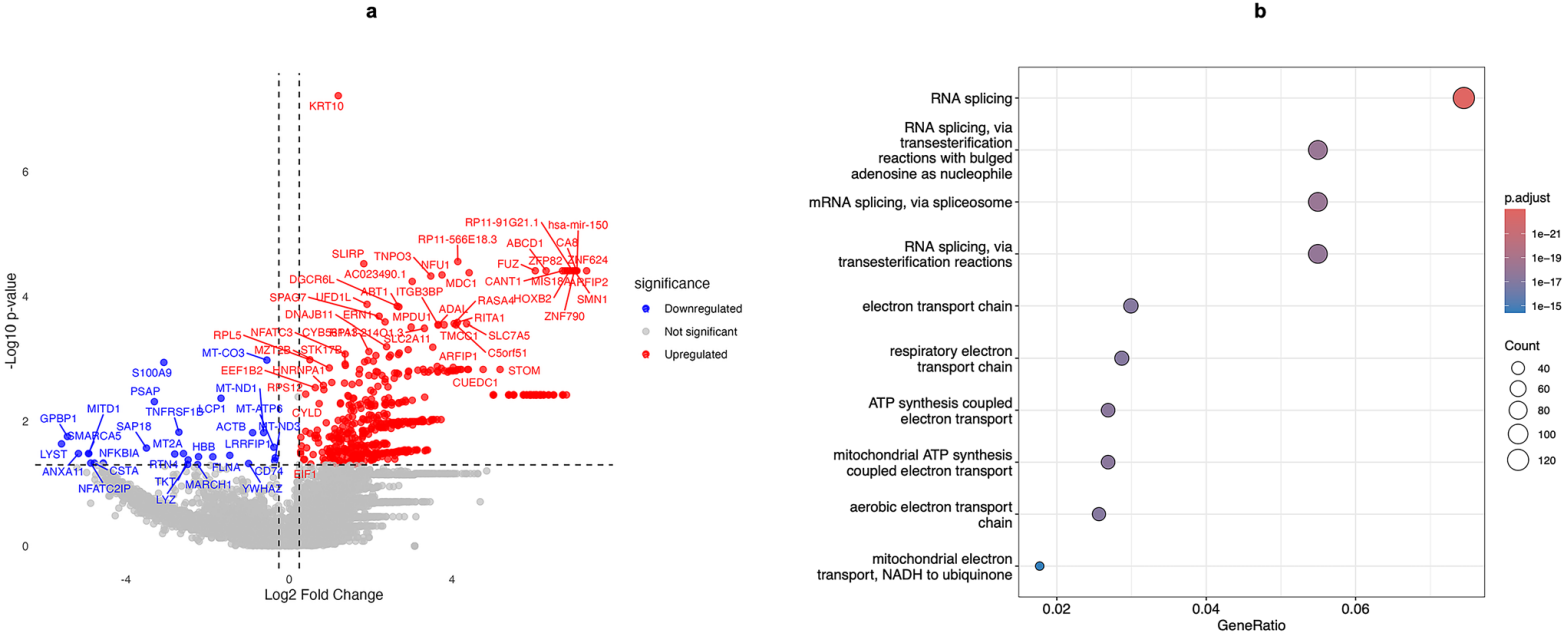
Differential expression and pathway enrichment in hybrid versus non-hybrid CTCs. **(a)** Volcano plot showing differentially expressed genes (DEGs) between hybrid CTCs and non-hybrid CTCs (epithelial and mesenchymal combined). Red and blue points indicate upregulated and downregulated genes in hybrid CTCs, respectively, based on |log₂ fold change|> 0.25 and adjusted *p* < 0.05. A total of 628 genes were significantly upregulated and 28 downregulated, with *KRT10* among the most significantly upregulated genes. **(b)** Dot plot of the top 10 enriched Gene Ontology (GO) biological processes among DEGs. Dot size represents the number of DEGs per pathway, dot colour corresponds to the adjusted p-value, and GeneRatio indicates the fraction of DEGs in each pathway relative to the total number of DEGs overlapping in any reference pathways. Enriched pathways reflect RNA metabolism, protein trafficking, and mitochondrial/energy-related processes in hybrid CTCs

Differential expression analysis comparing hybrid CTCs to non-hybrid CTCs (epithelial and mesenchymal combined) considered 14,242 genes tested in the analysis, of which 656 were significantly differentially expressed, including 628 upregulated and 28 downregulated genes in hybrid CTCs (|log₂ fold change| > 0.25, *p* < 0.05). Notably, *KRT10* was among the most significantly upregulated genes (Fig. 6a).

Pathway enrichment analysis of the DEGs identified 20 pathways enriched in hybrid CTCs (Table S4), with the top 10 pathways shown in Fig. 6b. The analysis revealed a predominance of RNA metabolism-related processes. The most significantly enriched pathways included RNA splicing, RNA splicing via transesterification reactions, and mRNA splicing via the spliceosome (all adjusted *p* < 10⁻¹⁸).

Additional enriched pathways were associated with mitochondrial energy production and electron transport, including ATP synthesis-coupled electron transport, mitochondrial ATP synthesis-coupled electron transport, respiratory electron transport chain, aerobic electron transport chain, and mitochondrial electron transport from NADH to ubiquinone (all adjusted *p* < 10⁻¹⁴). Collectively, these findings suggest that hybrid CTCs are characterised by transcriptional programmes associated with RNA processing, mitochondrial function, and cellular energy metabolism.

## Discussion

In this study, we explored the heterogeneity of CTCs in CRC using scRNA-seq on CRC PBMC fractions. Instead of pre-selecting cells using epithelial markers, we performed transcriptome-wide profiling of all captured cells. While cell capture was not dependent on marker-based enrichment, downstream CTC classification remained marker-guided and was based on epithelial, mesenchymal, and immune markers reported in the literature (Table S1). To improve cell-type annotation, CRC samples were batch-corrected together with externally sourced healthy PBMC datasets, providing a transcriptional reference for abundant immune cell populations within the dataset. Prior to batch correction, cells clustered predominantly according to sample of origin, indicating the presence of batch effects between datasets (Fig S6a). Following integration, improved sample mixing was observed, while centroid distance and LISI analyses supported effective integration without compromising the broader biological structure (Fig. 2a-c). These analyses supported the use of the integrated dataset for cell-type annotation and CTC identification.

CTCs were identified in one of the four patients analysed (CRC_1), highlighting the challenges associated with detecting CTCs in circulation. Several technical factors may have contributed to reduced CTC detectability, including PBMC isolation, CD45 depletion, and transcript dropout associated with 3′ gene expression [19, 33]. Consequently, we cannot conclude that CTCs were truly absent in CRC_2–CRC_4; however, these samples were considered CTC-negative within the context of the present analysis. Interestingly, CRC_1 the only sample in which CTCs were detected, had Stage II disease (Table 1). One study similarly reported detectable CTCs across disease stages, including a higher proportion of CTC-positive Stage II CRC cases compared with other stages, although this association did not reach statistical significance [34].

Beyond transcriptome-based approaches, several alternative strategies have been used to identify and enrich CTCs in CRC and other solid cancers. Common approaches include epithelial marker–based platforms such as CellSearch and immunomagnetic capture using EpCAM-specific antibodies [35, 36]. While these methods have demonstrated clinical utility, they are known to under-detect CTCs undergoing EMT because of reduced epithelial marker expression [35, 37]. Consistent with this, *EPCAM* expression was limited within our dataset, aligning with previous reports describing reduced epithelial-marker expression in CTCs during haematogenous dissemination [38–40]. Consequently, approaches relying solely on EpCAM for CTC identification may fail to capture the full spectrum of CTC phenotypes. Physical enrichment strategies based on size and deformability, including MetaCell filtration, overcome dependence on surface markers but remain limited by loss of smaller CTCs and co-enrichment of leukocytes [41, 42]. Transcriptomic profiling therefore provides an opportunity to characterise the phenotypic diversity and cellular plasticity underlying metastatic dissemination.

Metastasis is a highly dynamic process that requires CTCs to exhibit cellular plasticity, a property often facilitated by EMT. In this study, we identified three CTC subpopulations: epithelial, hybrid, and mesenchymal, representing different positions along the EMT continuum. Here, trajectory inference supported a continuous EMT spectrum, with epithelial CTCs occupying early pseudotime, hybrid CTCs intermediate pseudotime, and mesenchymal CTCs late pseudotime (Fig. 5a). These findings are consistent with previous single-cell trajectory studies in breast cancer and skin squamous cell carcinoma CTCs, which also reported a continuous EMT spectrum rather than discrete cell states [29, 43].

To examine changes in canonical epithelial and mesenchymal marker expression along the inferred trajectory, their expression was visualised in a scaled heatmap ordered by pseudotime (Fig. 5b). *KRT10* showed high expression in epithelial CTCs, was retained in hybrid CTCs, and progressively decreased towards mesenchymal CTCs, whereas *MALAT1* showed the opposite pattern, increasing from early to late pseudotime (Fig. 5b). Together, these findings support a continuous transcriptional spectrum linking epithelial, hybrid, and mesenchymal CTC states, consistent with the concept of partial EMT driving cellular plasticity and metastatic competence in CTCs [12, 29]. Cells in a hybrid E/M state or undergoing partial EMT have a higher propensity to acquire stem cell-like characteristics than fully epithelial or mesenchymal cells, suggesting a “stemness window” between differentiated states that may facilitate metastatic dissemination and survival in circulation [44, 45].

Visualisation of differentially expressed genes between the hybrid and non-hybrid (epithelial, and mesenchymal) CTCs using a heatmap revealed a distinct transcriptional pattern unique to hybrid CTCs. Differential expression analysis, guided by these visual patterns, identified 41 genes consistently upregulated in hybrid CTCs compared with purely epithelial or purely mesenchymal populations (Fig. 4). Among the 41 genes, *MALAT1* was strongly expressed in hybrid and mesenchymal CTCs but absent in epithelial CTCs, indicating its association with EMT and cellular plasticity in CRC. In contrast, *KRT10*, an epithelial marker, was expressed in epithelial and hybrid CTCs, highlighting the dual identity of hybrid cells. Together, these marker patterns support the concept of hybrid CTCs exhibiting both epithelial and mesenchymal features. To our knowledge, the specific expression pattern of *KRT10* and *MALAT1* in these CTC subpopulations has not been previously reported in CRC CTCs, suggesting that this signature warrants further validation in larger cohorts.

The distinct transcriptional profile observed in hybrid CTCs extended beyond individual genes and was associated with coordinated biological programmes. For pathway enrichment analysis, all 14,242 genes were included to capture coordinated transcriptional changes across gene networks that may not be evident when considering only the most significantly differentially expressed genes. This approach follows the principles of gene set enrichment analysis (GSEA), which evaluates gene sets across the full ranked dataset rather than imposing arbitrary gene-selection cut-offs, enabling detection of subtle but biologically meaningful pathway-level signals [46]. PEA revealed convergence on RNA metabolism and processing, protein transport and Golgi vesicle trafficking, cytoskeletal organisation, DNA repair and cell-cycle regulation, and mitochondrial energy production. Similar biological programmes have been reported in other cancers. For example, metabolic plasticity has been shown to enhance CTC fitness and metastatic competence in prostate cancer [47], while increased mitochondrial function and oxidative phosphorylation support CTC survival under shear and oxidative stress in breast cancer [48]. Likewise, enhanced DNA-damage responses may enable CTCs to withstand genotoxic and replicative stress [49], whereas vesicle trafficking has been implicated in protecting and promoting disseminating tumour cells, consistent with our enrichment for protein transport and Golgi-trafficking pathways [50]. Together, these findings suggest that hybrid CTCs engage coordinated metabolic, structural, and regulatory adaptations that may support survival in circulation and enhance metastatic competence in CRC.

A key limitation of this study is that CTCs were detected in only one of four patients (CRC_1). Consequently, all differential expression, pathway enrichment, and trajectory analyses were based on CTCs from a single patient. Therefore, inter-patient heterogeneity could not be assessed, and the findings should be considered exploratory and may not fully represent CTC biology across CRC. An additional limitation is that healthy control datasets were obtained from external sources rather than being processed alongside CRC samples. Although centroid distance and LISI analyses indicated improved sample mixing while retaining broader biological structure, disease status and dataset origin were fully confounded. Consequently, the external control datasets were used primarily for cell-type annotation and to provide a reference for abundant immune populations within the integrated dataset, rather than as direct biological comparators for downstream CTC analyses. Future studies incorporating matched healthy control samples processed under identical experimental conditions would provide a more robust reference. Finally, the small number of hybrid CTCs (*n* = 28) relative to non-hybrid cells (*n* = 237) resulted in unbalanced group sizes for differential expression analysis, which may affect statistical interpretation. Although our approach represents a broader strategy than previous methods relying on only a few markers to define CTCs, it remains limited by current knowledge and the lack of a fully standardised CTC definition in CRC.

## Conclusion

This study explored CTC heterogeneity in CRC using single-cell transcriptomics. Within the CTC-positive sample analysed, epithelial, hybrid, and mesenchymal CTC states were identified, with hybrid CTCs exhibiting distinct transcriptional features. These findings provide preliminary insight into the transcriptional diversity of CRC CTCs and highlight the potential of single-cell transcriptomic approaches to identify CTC subpopulations that may be missed by conventional marker-based methods. Further studies in larger, systematically powered cohorts are required to evaluate the reproducibility and prevalence of these CTC subpopulations and to determine their biological and clinical relevance across colorectal cancer patients and disease stages.

## Supplementary Information

Below is the link to the electronic supplementary material.

Supplementary Material 1

## Data Availability

The data presented in this study are available upon reasonable request. Access requires approval from the Dunedin Colorectal Cohort, and data will be shared once the corresponding authors receive confirmation of this approval.
