# Supplementary material for: Single-cell RNA sequencing of circulating tumour cells in colorectal cancer
Source: Mol Biol Rep. 2026 Jul 21;53(1):1224. doi: 10.1007/s11033-026-12427-0 (PMC13388743; doi:10.1007/s11033-026-12427-0)
Supplement: Supplementary file 1 — Supplementary Material 1 [file 11033_2026_12427_MOESM1_ESM.docx]

**Single-Cell RNA Sequencing of Circulating Tumour Cells in Colorectal Cancer**

Sai Shyam Vasantharajan^1^, Priyadarshana Ajithkumar^1^, Kit Moloney-Geany^2^, Hannah O’Neill^5^, Euan J. Rodger^1^, Sharon Pattison ^1,3^, Gregory Gimenez^1*^and Aniruddha Chatterjee^1,4*#^

1. Department of Pathology and Molecular Medicine, Faculty of Medicine, University of Otago, Ōtākau Whakaihu Waka, PO Box 56, Hercus Building, Cnr Great King & Hanover Streets, Otago, 9054, Dunedin, New Zealand

2. Department of Biochemistry, Faculty of Biomedical Sciences, University of Otago, Ōtākau Whakaihu Waka, PO Box 56, 710 Cumberland St, 9016, Dunedin, New Zealand

3. Health New Zealand Te Whatu Ora – Capital, Coast and Hutt Valley, Private Bag 7902, 49 Riddiford Street, Newtown, Wellington, 6242, Wellington, New Zealand

4. School of Health Sciences and Technology, UPES University, PO Bidholi, Knowledge Acres, Via Premnagar, Uttarakhand, 248007, Dehradun, India

5. Number 1 Fertility, Melbourne, Australia

* Joint Senior Author

# Correspondence: [aniruddha.chatterjee@otago.ac.nz](mailto:aniruddha.chatterjee@otago.ac.nz)

**Supplementary Figures**


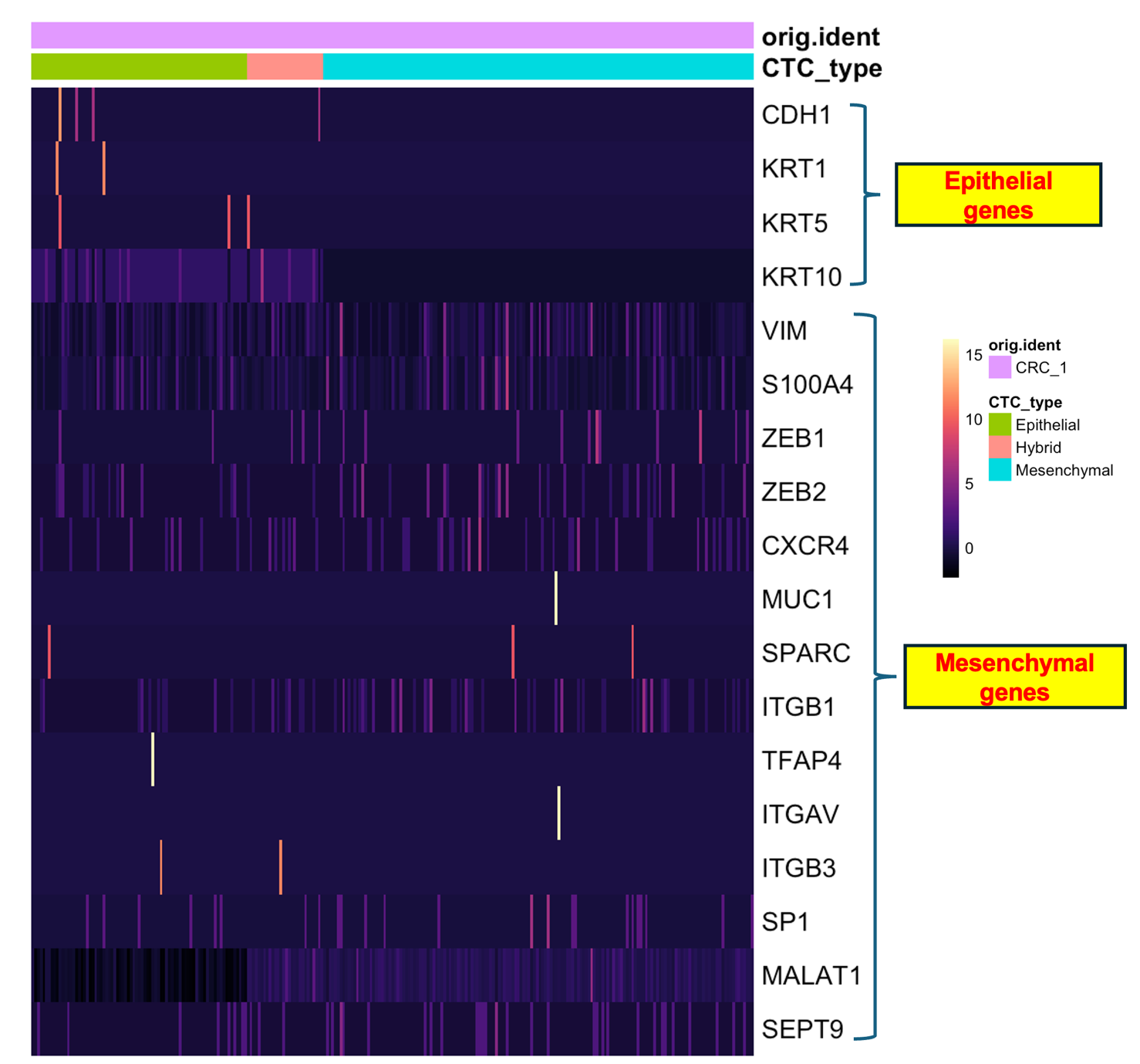


**Fig S1. Expression of epithelial and mesenchymal marker genes across CTC subpopulations.** Heatmap showing the expression of selected epithelial and mesenchymal marker genes across epithelial, hybrid, and mesenchymal CTCs. Rows represent marker genes and columns represent individual cells. CTC subpopulations are annotated at the top of the heatmap, with epithelial CTCs shown in green, hybrid CTCs in pink, and mesenchymal CTCs in light blue. Only marker genes detected in the dataset were included, with no additional weighting or scaling beyond standard normalisation. Most markers show broadly consistent expression across subpopulations, while select epithelial markers including *CDH1*, *KRT1*, and *KRT5*, and mesenchymal markers including *MUC1*, *SPARC*, *TFAP4*, *ITGAV*, and *ITGB3*, display sparse high expression in subsets of cells, highlighting intra-population heterogeneity.


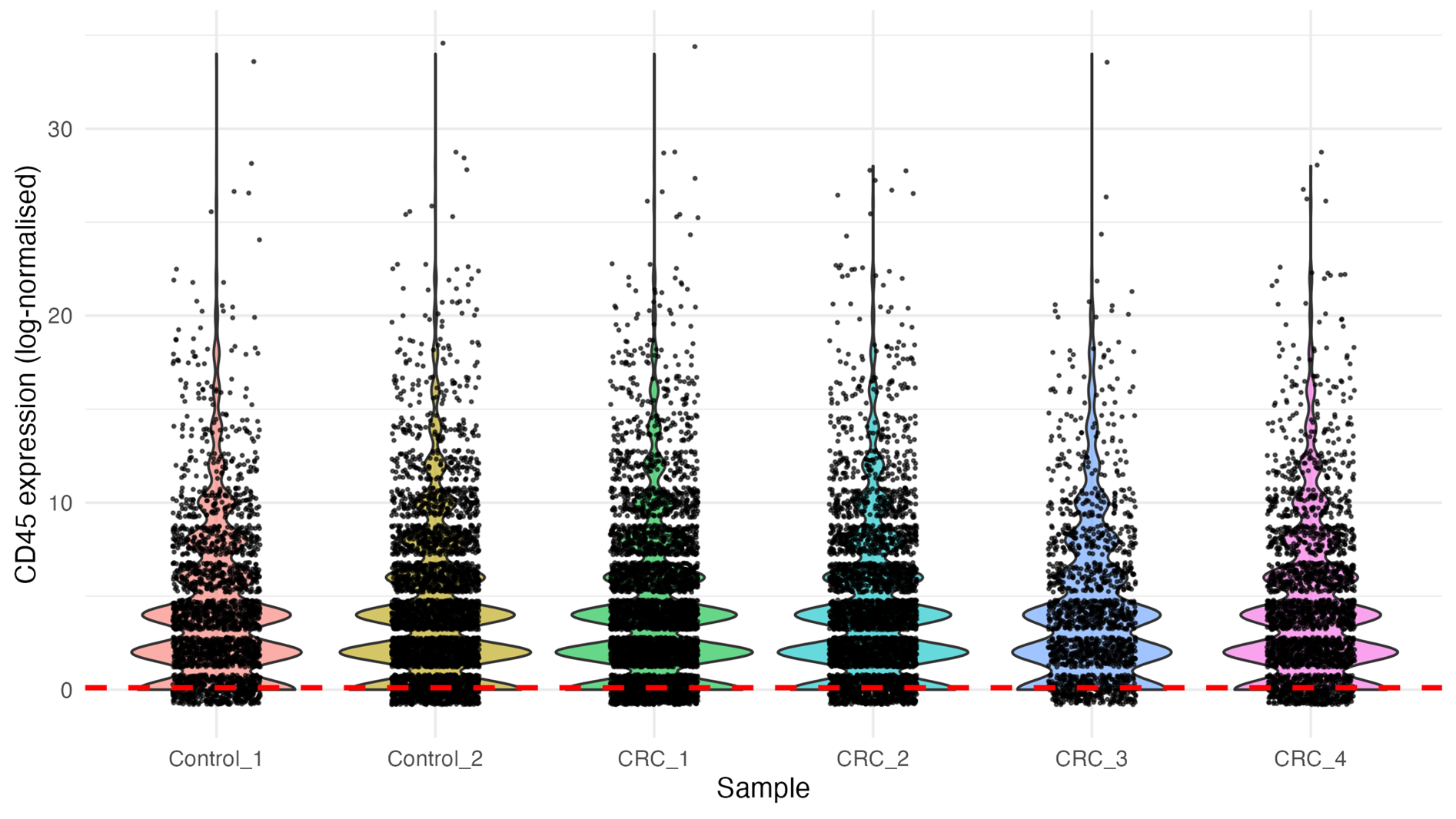


**Fig S2. Determination of CD45 threshold for CTC classification.** Violin plots show the distribution of log-normalised *CD45* expression across individual cells from CRC and control samples. A red dashed horizontal line indicates the selected CD45 threshold (≤ 0.1), chosen based on inspection of expression distributions to capture a clear low-expression population while allowing for low-level technical noise. This threshold was applied as one of the criteria for CTC classification and downstream analyses.


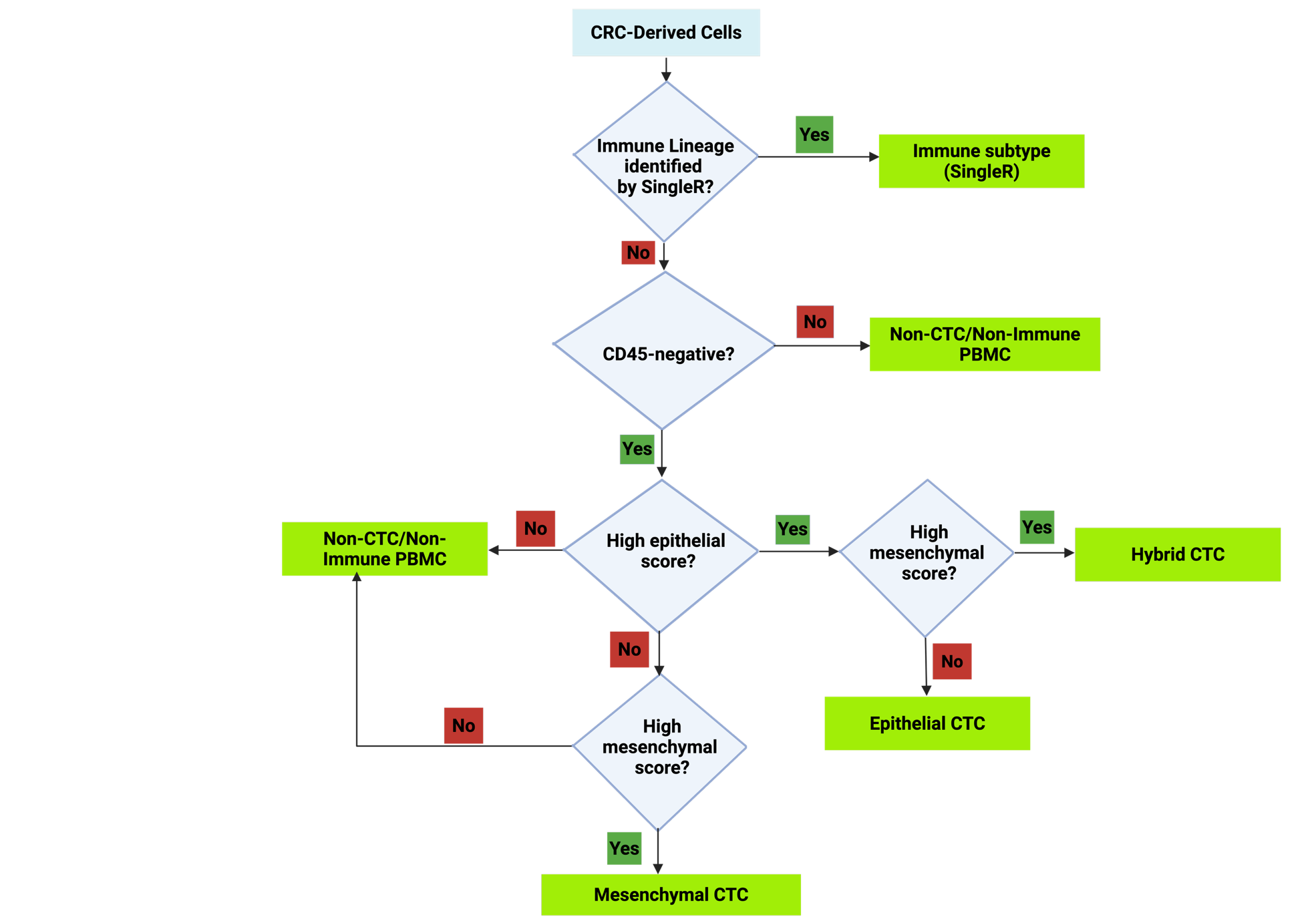


**Fig S3.** **Hierarchical annotation strategy used for immune cell and CTC classification.** CRC-Derived cells were first assessed for immune lineage identity using SingleR. Cells assigned to canonical immune lineages were classified as immune subtypes. Remaining cells were evaluated for CD45-negative status (CD45 ≤ 0.1) and classified according to epithelial and mesenchymal scores. Cells with high epithelial scores alone were classified as epithelial CTCs, cells with high mesenchymal scores alone as mesenchymal CTCs, and cells with both high epithelial and mesenchymal scores as hybrid CTCs. Cells not meeting the criteria for immune or CTC classification were assigned as non-CTC/non-immune PBMCs. This hierarchical framework was used to generate the final CombinedLabel annotation for downstream analyses. **Diamonds indicate decision points, grey rectangles indicate processing or input steps, and green coloured rectangles indicate final cell classifications.**


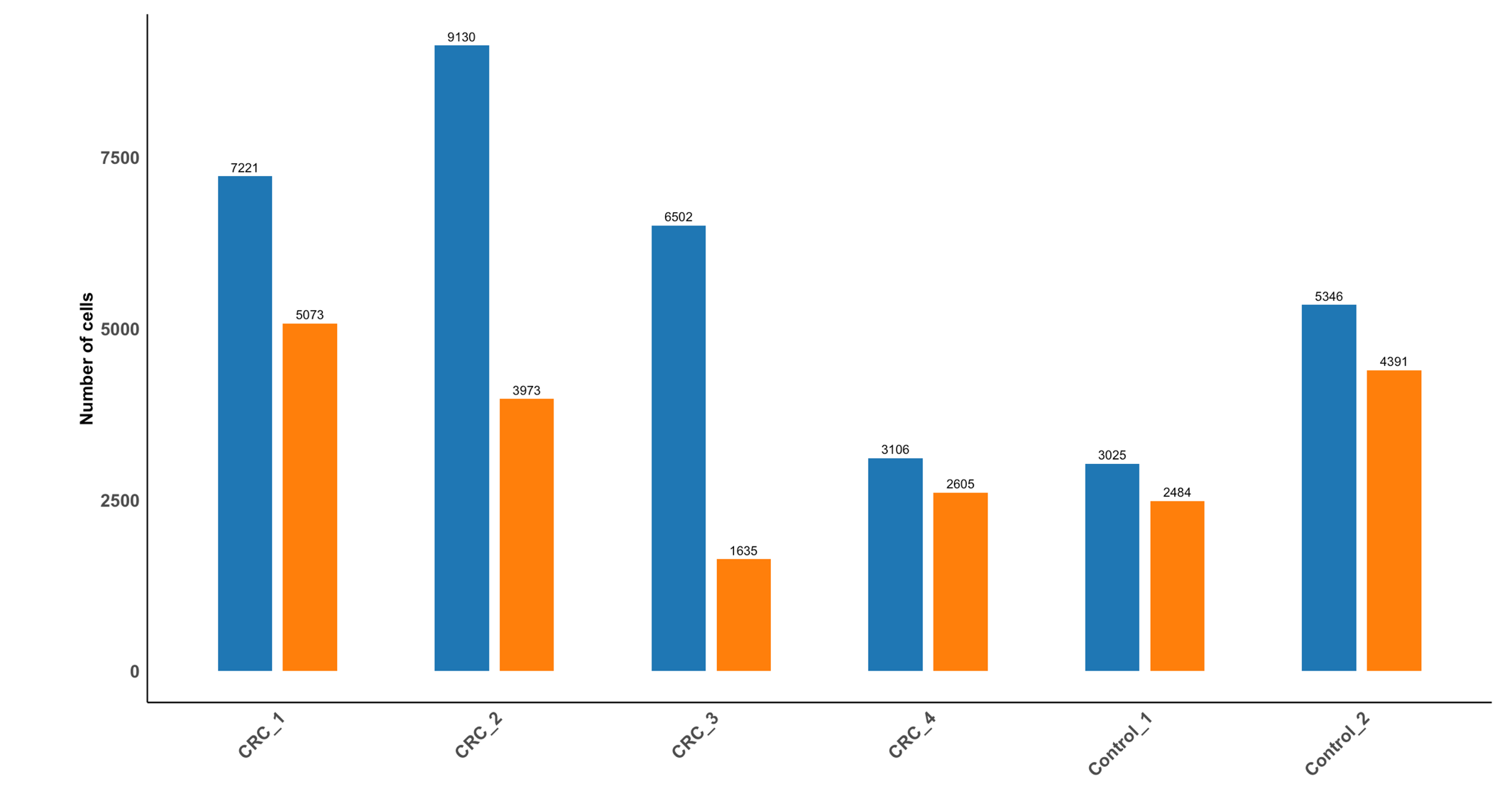


**Fig S4. Cell numbers before and after quality-control filtering.** Bar graph showing the number of cells detected in each sample before (blue bar) and after applying quality-control filters (orange bar). Pre-filter counts were derived from the initial gene–count matrices and include empty droplets, cells with low gene content, cells with high mitochondrial RNA content, and potential doublets. After filtering, these low-quality or artefactual events and doublets were removed, yielding a refined set of high-quality cells used for downstream integration, clustering, and cell-type analyses.

**
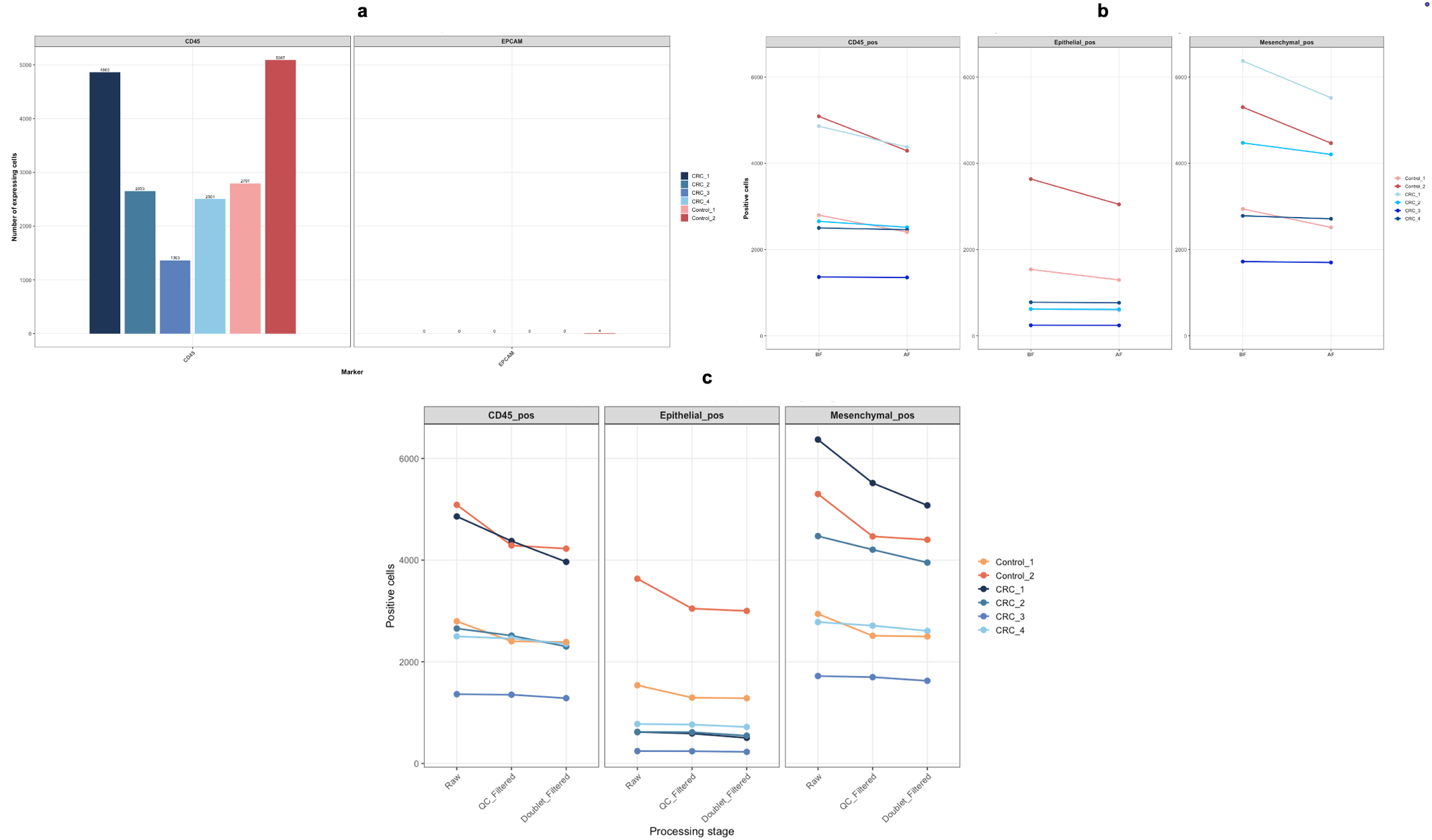
**

**Fig S5. Assessment of epithelial, mesenchymal, and CD45-associated populations across preprocessing stages.** **(a)** Number of CD45-positive and EpCAM-positive cells detected across all samples in the raw count matrices prior to quality-control filtering and batch correction. Cells were considered positive when expression was greater than zero. **(b)** Comparison of epithelial, mesenchymal, and CD45-associated populations before (BF) and after (AF) application of the 20% mitochondrial filtering threshold. The y-axis represents the number of positive cells detected across samples. **(c)** Tracking of epithelial-, mesenchymal-, and CD45-associated populations across sequential preprocessing stages, including raw data, QC-filtered data, and doublet-filtered data. The x-axis represents preprocessing stage, and the y-axis represents the number of positive cells detected across samples.


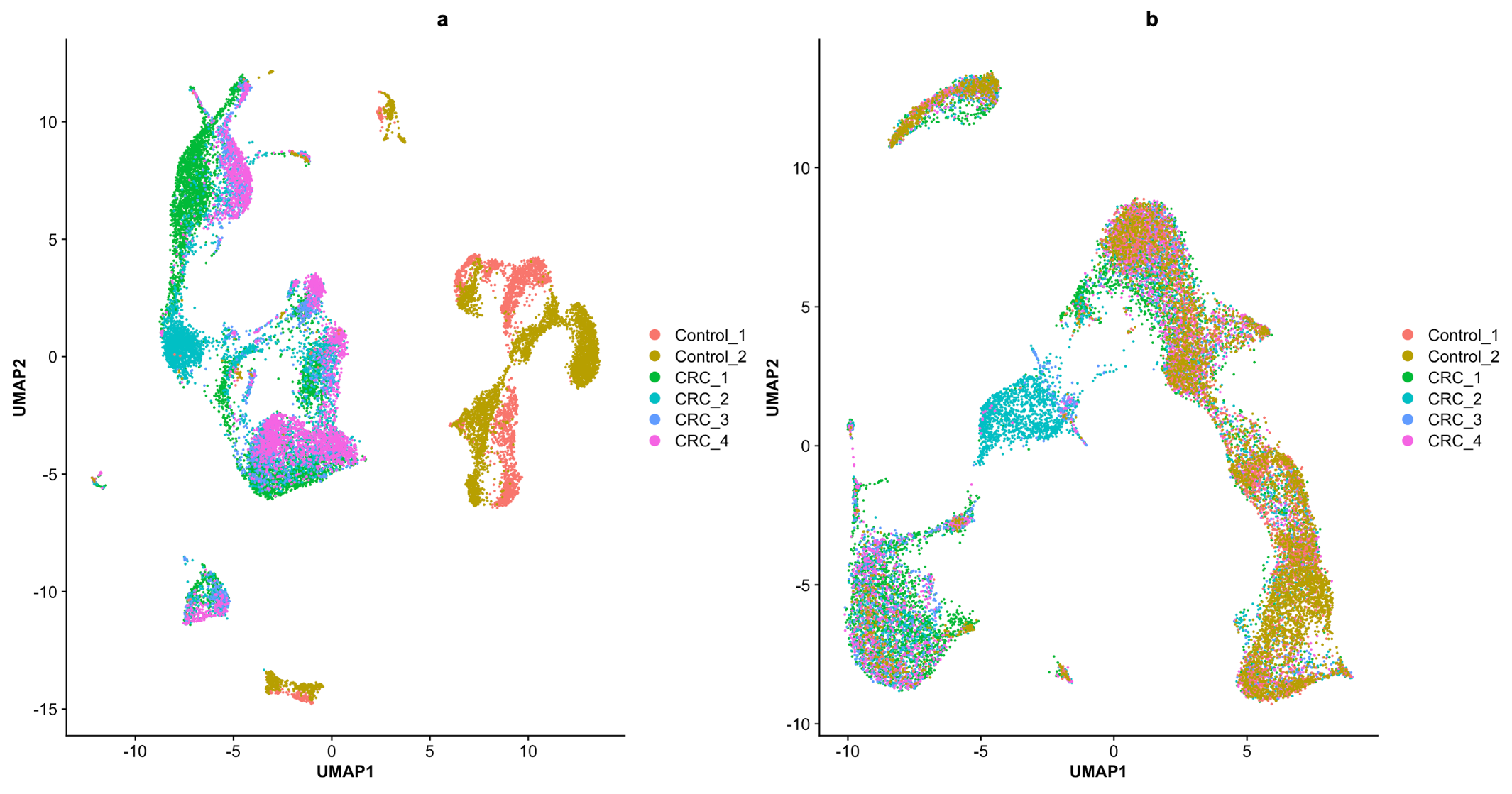


**Fig S6. Assessment and correction of batch effects in scRNA-seq data.** **(a)** UMAP projection of all cells prior to integration, coloured by sample of origin. Cells cluster primarily by dataset, indicating the presence of batch effects between in-house CRC samples and external healthy controls. **(b)** UMAP projection following Seurat integration. Cells exhibit improved mixing across samples following integration while maintaining transcriptionally distinct cell populations.


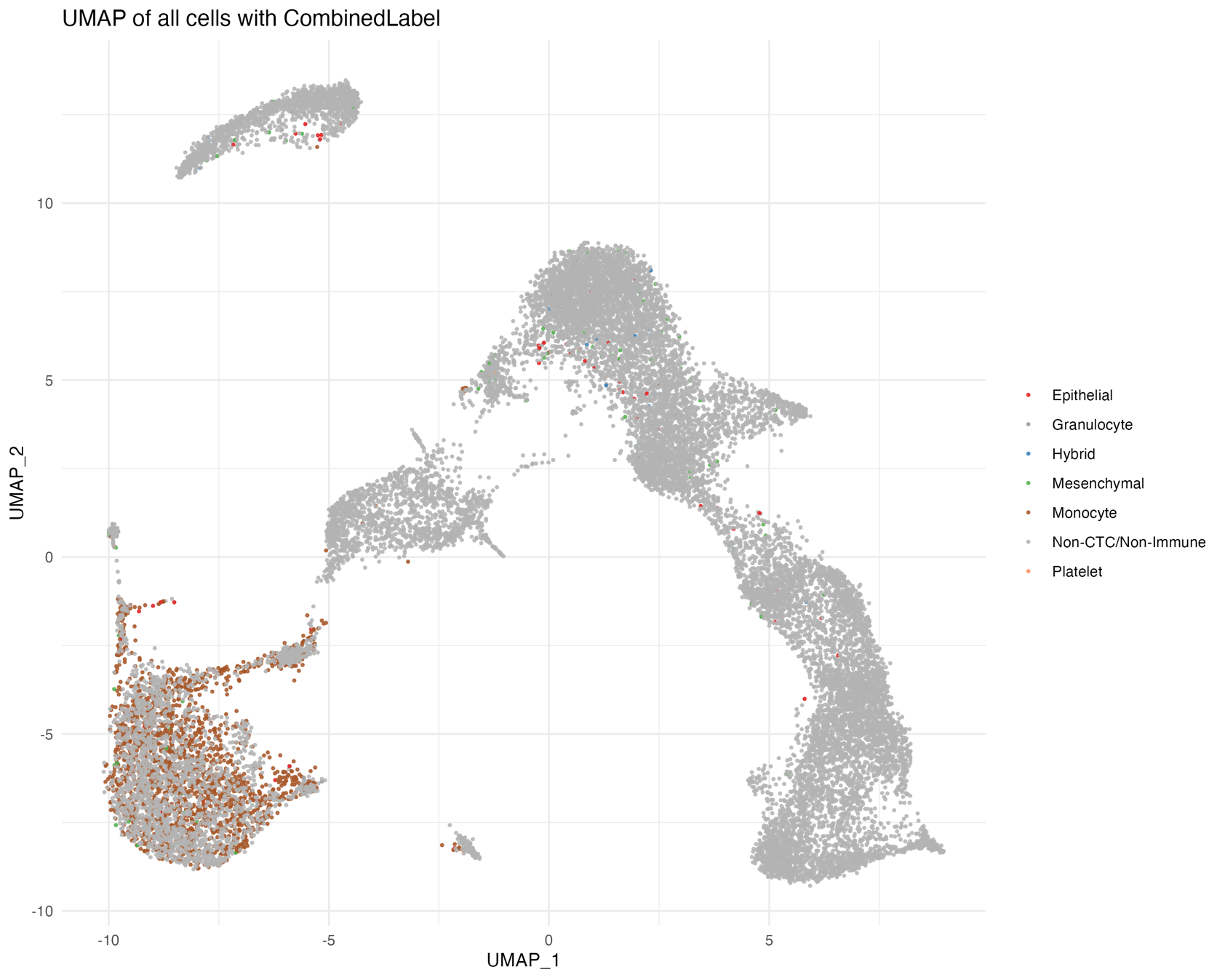


**Fig S7. UMAP visualisation of all cells in the integrated dataset.** Each point represents a single cell, positioned according to transcriptional similarity. Cells are coloured by their assigned label, including epithelial CTCs (red dots), mesenchymal CTCs (light green dots), hybrid CTCs (light blue dots), monocytes (brown dots), granulocytes and non-CTC or non-immune cells (grey dots) and platelets (Orange dots). This plot shows the overall cellular composition and the distinct clustering patterns of each population.


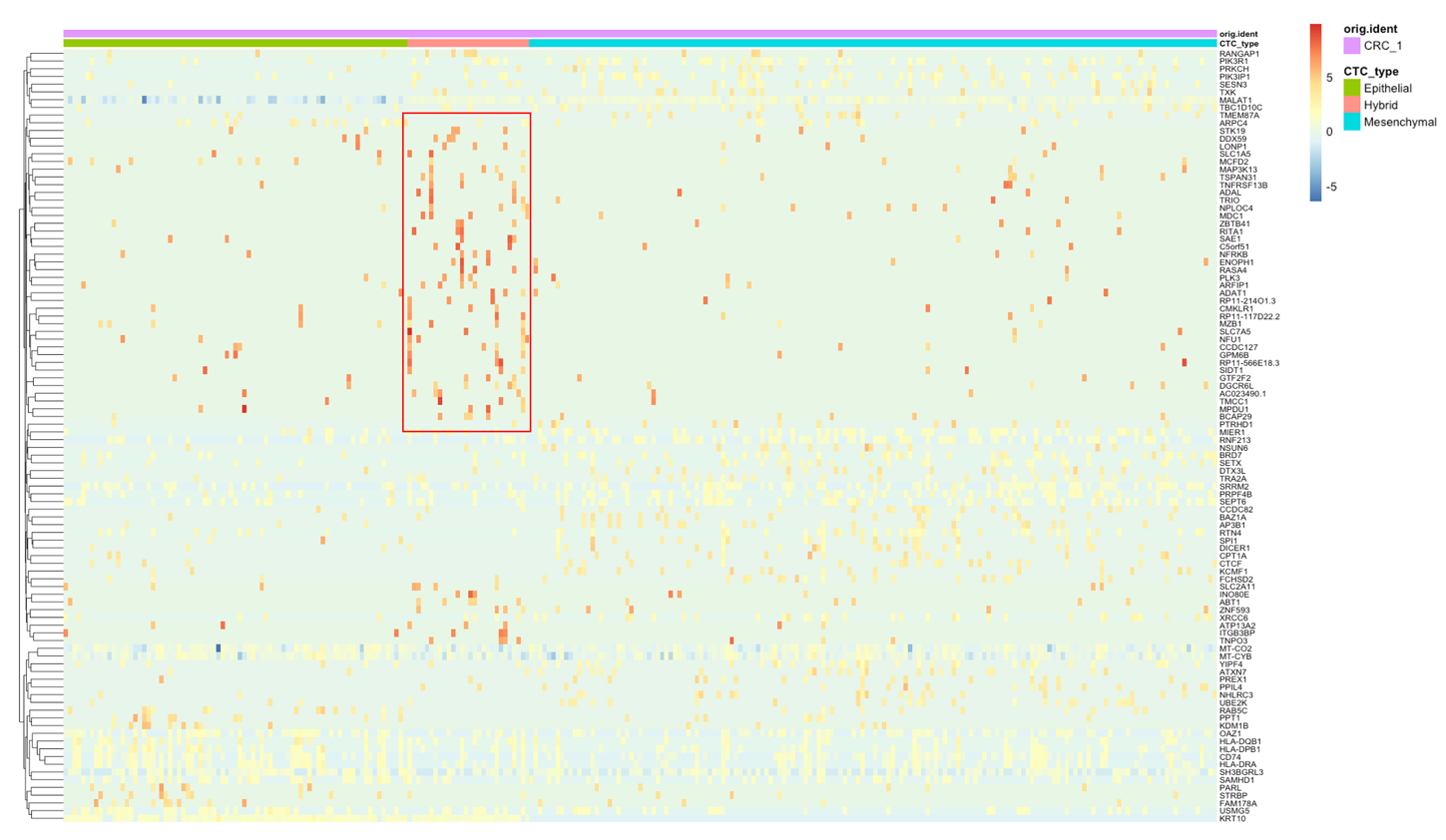


**Fig S8. Heatmap of the top 100 differentially expressed genes across epithelial, mesenchymal, and hybrid CTCs.** Heatmap of 100 differentially expressed genes (DEGs) between epithelial (green bar), mesenchymal (blue bar) and hybrid CTCs (pink bar) was generated**.** Each row represents a gene and each column a cell. Among the 100 DEGs, 41 genes were upregulated in hybrid CTCs compared with both epithelial and mesenchymal CTCs. 39 of these genes formed a coherent expression block and were highlighted with a red box. Two additional genes, *KRT10* and *MALAT1*, showed distinct expression patterns, with *KRT10* elevated in epithelial and hybrid CTCs and *MALAT1* elevated in hybrid and mesenchymal CTCs, reflecting the mixed transcriptional characteristics of hybrid CTCs; these two genes were not boxed due to their non-contiguous positioning in the ranked list.

**Supplementary Tables**

**Table S1. List of CTC markers chosen for CTC detection.**

| Type of CTC Markers | Genes | References |
| --- | --- | --- |
| Epithelial | 1. *EPCAM*  2. *KRT1*  *3. KRT2*  *4. KRT3*  *5. KRT4*  *6. KRT5*  *7. KRT6*  *8. KRT7*  *9. KRT8*  *10. KRT9*  *11. KRT10*  12. *KRT 12*  13. *KRT13*  *14. KRT14*  *15 .KRT16*  *16. KRT17*  *17. KRT18*  *18. KRT19*  *19. KRT20*  20. *CLDN4*  21. *CDH1*  22. *AGR2*  23. *CLDN3*  24. *TROP2* | [1-3] |
| Mesenchymal | 1. *VIM* 2. *FN1* 3. *SNAI1* 4. *S100A4* 5. *ZEB1* 6. *ZEB2* 7. *SNAI1* 8. *CXCR4* 9. *CDH2* 10. *VCAM1* 11. *MUC1* 12. *SPARC* 13. *PLS3* 14. *ITGB1* 15. *TFAP4* 16. *ITGAV* 17. *ITGB3* 18. *SP1* 19. *CEACAM5* 20. *FGFR3* 21. *SERPINE1F* 22. *FOXC1* 23. *MALAT1* 24. *SEPT9* | [1-5] |

**Abbreviations: *EpCAM* -** Epithelial Cell Adhesion Molecule, ***KRT 1-20 -*** Cytokeratin 1-20, ***CLDN4* -** Claudin 4, ***CDH1-*** Cadherin-1, ***AGR2* -** Anterior gradient protein 2 homolog, ***CLDN3* -** Claudin 3, ***TROP2*** - Trophoblast Cell Surface Antigen 2, ***VIM* –** Vimentin, ***FN1*-** Fibronectin 1**, *ZEB1-*** Zinc Finger E-Box Binding Homeobox 1,  ***ZEB2* -** Zinc Finger E-Box Binding Homeobox 2, ***SNAI1* -** Snail Family Transcriptional Repressor 1, ***CXCR4* -** C-X-C Motif Chemokine Receptor 4, ***CDH2* -** Cadherin 2, ***VCAM1* -** Vascular Cell Adhesion Molecule 1, ***MUC1* -** Mucin 1, ***SPARC* -** Secreted Protein Acidic And Cysteine Rich, ***PLS3* -** Plastin 3, ***ITGB1* -** Integrin beta-1, ***ITGB3 -*** Integrin beta-3, ***TFAP4* -** Transcription Factor AP-4, ***ITGAV* -** Integrin Subunit Alpha V, ***SP1* -** Specific protein 1, ***CEACAM5* -** Carcinoembryonic antigen-related cell adhesion molecule 5, ***FGFR3* -** *F*ibroblast growth factor receptor 3, ***SERPINE1F* -** Serpin Family F Member 1, ***FOXC1* -** Forkhead Box C1,  ***MALAT1* -** Metastasis Associated Lung Adenocarcinoma Transcript 1, ***SEPT9* -** Septin 9

**Table S2.** Quantile thresholds showing the number of epithelial and mesenchymal CTCs

retained at each filtering level.

| Quantile | Epithelial cells | Mesenchymal cells |
| --- | --- | --- |
| 0.50 | 506 | 2479 |
| 0.55 | 506 | 2233 |
| 0.60 | 506 | 2016 |
| 0.65 | 506 | 1755 |
| 0.70 | 506 | 1507 |
| 0.75 | 506 | 1261 |
| 0.80 | 506 | 980 |
| 0.85 | 506 | 756 |
| 0.90 | 506 | 496 |
| 0.95 | 34 | 249 |

**Table S3.** Expression levels of the different epithelial and mesenchymal markers

used in this study for CTC detection

| Gene | Type | Mean expression (log normalised counts) |
| --- | --- | --- |
| *EPCAM* | Epithelial | Not detected |
| *CLDN4* | Epithelial | Not detected |
| *CDH1* | Epithelial | 0.038 |
| *AGR2* | Epithelial | Not detected |
| *CLDN3* | Epithelial | Not detected |
| *KRT1* | Epithelial | 0.015 |
| *KRT2* | Epithelial | Not detected |
| *KRT3* | Epithelial | Not detected |
| *KRT4* | Epithelial | Not detected |
| *KRT5* | Epithelial | 0.023 |
| *TROP2* | Epithelial | Not detected |
| *KRT6* | Epithelial | Not detected |
| *KRT7* | Epithelial | Not detected |
| *KRT8* | Epithelial | Not detected |
| *KRT9* | Epithelial | Not detected |
| *KRT10* | Epithelial | 0.838 |
| *KRT12* | Epithelial | Not detected |
| *KRT13* | Epithelial | Not detected |
| *KRT14* | Epithelial | Not detected |
| *KRT16* | Epithelial | Not detected |
| *KRT17* | Epithelial | Not detected |
| *KRT18* | Epithelial | Not detected |
| *KRT19* | Epithelial | Not detected |
| *KRT20* | Epithelial | Not detected |
| *VIM* | Mesenchymal | 3.313 |
| *FN1* | Mesenchymal | Not detected |
| *S100A4* | Mesenchymal | 3.909 |
| *ZEB1* | Mesenchymal | 0.136 |
| *ZEB2* | Mesenchymal | 0.566 |
| *SNAI1* | Mesenchymal | Not detected |
| *CXCR4* | Mesenchymal | 0.491 |
| *CDH2* | Mesenchymal | Not detected |
| *VCAM1* | Mesenchymal | Not detected |
| *MUC1* | Mesenchymal | 0.008 |
| *SPARC* | Mesenchymal | 0.023 |
| *PLS3* | Mesenchymal | Not detected |
| *ITGB1* | Mesenchymal | 0.747 |
| *TFAP4* | Mesenchymal | 0.008 |
| *ITGAV* | Mesenchymal | 0.008 |
| *ITGB3* | Mesenchymal | 0.015 |
| *SP1* | Mesenchymal | 0.196 |
| *CEACAM5* | Mesenchymal | Not detected |
| *FGFR3* | Mesenchymal | Not detected |
| *SERPINE1F* | Mesenchymal | Not detected |
| *FOXC1* | Mesenchymal | Not detected |
| *MALAT1* | Mesenchymal | 179.736 |
| *SEPT9* | Mesenchymal | 0.317 |

**Table S4** Gene ontology enrichment of differentially expressed genes in hybrid CTCs highlighting RNA processing, protein trafficking, and energy metabolism pathways.

| ID | Description | GeneRatio | Count | RichFactor | FoldEnrichment | zScore | p-value | p.adjust | qvalue |
| --- | --- | --- | --- | --- | --- | --- | --- | --- | --- |
| GO:0008380 | RNA splicing | 122/1638 | 122 | 0.248979592 | 2.866761357 | 12.91239344 | 1.79E-27 | 1.01E-23 | 8.09E-24 |
| GO:0000377 | RNA splicing, via transesterification reactions with bulged adenosine as nucleophile | 90/1638 | 90 | 0.261627907 | 3.012394582 | 11.61698052 | 4.17E-22 | 7.81E-19 | 6.28E-19 |
| GO:0000398 | mRNA splicing, via spliceosome | 90/1638 | 90 | 0.261627907 | 3.012394582 | 11.61698052 | 4.17E-22 | 7.81E-19 | 6.28E-19 |
| GO:0000375 | RNA splicing, via transesterification reactions | 90/1638 | 90 | 0.25862069 | 2.977769357 | 11.48452616 | 9.86E-22 | 1.38E-18 | 1.11E-18 |
| GO:0042773 | ATP synthesis coupled electron transport | 44/1638 | 44 | 0.44 | 5.066178266 | 12.57316122 | 4.63E-21 | 4.32E-18 | 3.48E-18 |
| GO:0042775 | mitochondrial ATP synthesis coupled electron transport | 44/1638 | 44 | 0.44 | 5.066178266 | 12.57316122 | 4.63E-21 | 4.32E-18 | 3.48E-18 |
| GO:0022904 | respiratory electron transport chain | 47/1638 | 47 | 0.408695652 | 4.705738706 | 12.29293196 | 9.19E-21 | 7.37E-18 | 5.93E-18 |
| GO:0019646 | aerobic electron transport chain | 42/1638 | 42 | 0.451612903 | 5.199889716 | 12.5215018 | 1.07E-20 | 7.39E-18 | 5.95E-18 |
| GO:0022900 | electron transport chain | 49/1638 | 49 | 0.392 | 4.513504274 | 12.15466883 | 1.19E-20 | 7.39E-18 | 5.95E-18 |
| GO:0006120 | mitochondrial electron transport, NADH to ubiquinone | 29/1638 | 29 | 0.557692308 | 6.421292383 | 12.07279095 | 6.49E-18 | 3.64E-15 | 2.93E-15 |
| GO:0006119 | oxidative phosphorylation | 49/1638 | 49 | 0.331081081 | 3.812081312 | 10.59188152 | 4.45E-17 | 2.27E-14 | 1.83E-14 |
| GO:0043484 | regulation of RNA splicing | 56/1638 | 56 | 0.291666667 | 3.358262108 | 10.1290398 | 1.68E-16 | 7.87E-14 | 6.33E-14 |
| GO:0050684 | regulation of mRNA processing | 46/1638 | 46 | 0.330935252 | 3.810402228 | 10.256185 | 4.14E-16 | 1.79E-13 | 1.44E-13 |
| GO:0000380 | alternative mRNA splicing, via spliceosome | 35/1638 | 35 | 0.406976744 | 4.685947128 | 10.56560588 | 9.52E-16 | 3.81E-13 | 3.07E-13 |
| GO:0006403 | RNA localization | 57/1638 | 57 | 0.274038462 | 3.155290223 | 9.639382401 | 1.96E-15 | 7.33E-13 | 5.90E-13 |
| GO:0022613 | ribonucleoprotein complex biogenesis | 99/1638 | 99 | 0.199596774 | 2.298165544 | 9.035699114 | 2.15E-15 | 7.52E-13 | 6.05E-13 |
| GO:0042776 | proton motive force-driven mitochondrial ATP synthesis | 30/1638 | 30 | 0.441176471 | 5.079724197 | 10.39377342 | 7.71E-15 | 2.55E-12 | 2.05E-12 |
| GO:0006457 | protein folding | 59/1638 | 59 | 0.25877193 | 2.979510743 | 9.27406286 | 1.06E-14 | 3.30E-12 | 2.66E-12 |
| GO:0045333 | cellular respiration | 73/1638 | 73 | 0.217261905 | 2.501562591 | 8.564858236 | 1.41E-13 | 4.15E-11 | 3.34E-11 |
| GO:0009060 | aerobic respiration | 67/1638 | 67 | 0.225589226 | 2.597443709 | 8.557643533 | 2.22E-13 | 6.23E-11 | 5.02E-11 |
| GO:0015986 | proton motive force-driven ATP synthesis | 30/1638 | 30 | 0.394736842 | 4.545016387 | 9.550046583 | 2.80E-13 | 7.45E-11 | 6.00E-11 |

**Column Descriptions:** GO term identifier, **Description:** Name of the GO biological process, **GeneRatio:** Fraction of input genes annotated to the GO term (genes in term / total input genes), **Count:** Number of input genes associated with the GO term, **RichFactor:** Ratio of input genes in the GO term to total genes annotated to the term in the background, **FoldEnrichment:** Enrichment score of the GO term relative to background expectation, **zScore:** Standardised measure of deviation from expected enrichment, **p-value:** Significance of enrichment before multiple testing correction, **p.adjust:** Adjusted p-value after multiple testing correction (e.g., Benjamini-Hochberg), **qvalue:** Alternative false discovery rate (FDR) estimate

**Supplementary Methods**

**Assessment of epithelial, mesenchymal, and CD45 cell populations across preprocessing stages**

To evaluate whether preprocessing and filtering steps influenced the representation of epithelial-associated cell populations within the dataset, additional analyses were performed across multiple preprocessing stages. Raw count matrices generated following CellRanger processing were first examined to quantify the number of EpCAM-positive and CD45-positive cells detected across all samples prior to downstream preprocessing. Cells were classified as EpCAM-positive or CD45-positive using a permissive expression threshold of greater than zero within the raw RNA count matrices.

As mitochondrial filtering represented one of the principal quality-control filtering steps applied during preprocessing, the effect of mitochondrial filtering on epithelial, mesenchymal, and CD45 cell populations was subsequently assessed. Cells expressing epithelial, mesenchymal, or CD45 marker panels were quantified before and after application of the 20% mitochondrial filtering threshold. Marker panels used for epithelial and mesenchymal classification were consistent with those applied during downstream cell-state scoring analyses.

Additional tracking analyses were then performed across sequential preprocessing stages, including raw data, QC-filtered data, and doublet-filtered data. For each preprocessing stage, the number of epithelial, mesenchymal, and CD45 cells was quantified across all samples using expression matrices derived from the RNA assay. Cells were considered positive when expression of at least one marker within the corresponding marker panel was greater than zero.

These analyses were performed to evaluate whether the low abundance of epithelial and EpCAM-positive populations observed during downstream analyses could be attributed predominantly to preprocessing and filtering procedures.

**Integration of CRC and healthy control datasets**

To minimise technical variation between the CRC samples and external healthy control datasets, the datasets were integrated using the Seurat anchor-based integration workflow. Prior to integration, each dataset was normalised using the NormalizeData function, and highly variable genes were identified using the variance-stabilising transformation (VST) method implemented in FindVariableFeatures. The top 3,000 variable features from each dataset were used for integration. Integration anchors representing biologically similar cell states across datasets were identified using FindIntegrationAnchors based on the first 20 principal components. Batch effects were subsequently corrected during dataset integration using IntegrateData, which generated an integrated expression matrix by aligning shared cellular populations across samples while preserving biological heterogeneity.

The integrated assay was then used for downstream dimensionality reduction and clustering analyses. Integrated expression values were scaled using ScaleData, followed by principal component analysis (RunPCA). Uniform Manifold Approximation and Projection (UMAP) was performed using the first 20 principal components for visualisation. Cell–cell similarities were computed using FindNeighbors, and clusters were identified using the Louvain algorithm implemented in FindClusters with a resolution parameter of 0.8.

**Assessment of Batch Correction and Dataset Integration**

Following dataset integration, additional analyses were performed to evaluate batch-correction performance between the externally sourced healthy control datasets and CRC samples. UMAP visualisation was first used to qualitatively assess sample mixing before and after batch correction. In addition, centroid distance analyses were performed in principal component analysis (PCA) space to quantify inter-sample separation before and after batch correction. Pairwise centroid distances were calculated between individual samples using principal component embeddings generated before and after Seurat integration. Distances were subsequently compared between Control–Control, CRC–CRC, and CRC–Control sample pairs to assess whether integration reduced technical separation while preserving broader biological structure.

Furthermore, integration Local Inverse Simpson's Index (iLISI) and cell-type LISI (cLISI) analyses were performed to assess batch mixing and preservation of broader biological structure following integration. For iLISI, values approaching 2 indicated improved batch mixing across the two integration batches, whereas cLISI values approaching 1 indicate greater preservation of transcriptionally distinct cell populations.

**Assessment of Marker-Level Expression Patterns**

Only marker genes detected in the dataset were included, and no additional weighting or scaling was applied. To qualitatively assess marker-level expression patterns underlying the composite scores, a scaled heatmap of epithelial and mesenchymal markers was generated across epithelial, hybrid, and mesenchymal CTCs (Fig S1). This visualisation was used as an interpretative checkpoint to inspect co-expression patterns and heterogeneity of individual markers across CTC categories, rather than as a formal validation step. Summed scoring was used throughout the study, consistent with prior single-cell EMT studies that apply unweighted marker aggregation to capture continuum-like transcriptional states.

**References**

1. Kozuka M, Battaglin F, Jayachandran P, Wang J, Arai H, Soni S, et al. Clinical significance of circulating tumor cell induced epithelial-mesenchymal transition in patients with metastatic colorectal cancer by single-cell RNA-sequencing. Cancers. 2021;13(19):4862.

2. Negishi R, Yamakawa H, Kobayashi T, Horikawa M, Shimoyama T, Koizumi F, et al. Transcriptomic profiling of single circulating tumor cells provides insight into human metastatic gastric cancer. Communications biology. 2022;5(1):20.

3. Tieng FYF, Baharudin R, Abu N, Mohd Yunos R-I, Lee L-H, Ab Mutalib N-S. Single cell transcriptome in colorectal cancer—current updates on its application in metastasis, chemoresistance and the roles of circulating tumor cells. Frontiers in pharmacology. 2020;11:135.

4. Cima I, Kong SL, Sengupta D, Tan IB, Phyo WM, Lee D, et al. Tumor-derived circulating endothelial cell clusters in colorectal cancer. Science translational medicine. 2016;8(345):345ra89-ra89.

5. Grillet F, Bayet E, Villeronce O, Zappia L, Lagerqvist EL, Lunke S, et al. Circulating tumour cells from patients with colorectal cancer have cancer stem cell hallmarks in ex vivo culture. Gut. 2017;66(10):1802-10.
